# Stimuli‐Responsive Materials for Biomedical Applications

**DOI:** 10.1002/adma.202507559

**Published:** 2025-08-13

**Authors:** Adriana Teixeira do Nascimento, Paul R. Stoddart, Toon Goris, Miriam Kael, Richard Manasseh, Karen Alt, Jurie Tashkandi, Byung Chul Kim, Simon E Moulton

**Affiliations:** ^1^ Department of Engineering Technologies School of Engineering Swinburne University of Technology Victoria 3122 Australia; ^2^ Iverson Health Innovation Research Institute Swinburne University of Technology Victoria 3122 Australia; ^3^ Aikenhead Centre for Medical Discovery St Vincent's Hospital Melbourne Victoria 3065 Australia; ^4^ School of Physics University of Melbourne Melbourne Victoria 3010 Australia; ^5^ Department of Mechanical & Product Design Engineering School of Engineering Swinburne University of Technology Victoria 3122 Australia; ^6^ NanoTheranostics Laboratory The School of Translational Medicine Faculty of Medicine Nursing and Health Sciences Monash University Victoria 3800 Australia; ^7^ Department of Advanced Components and Materials Engineering Sunchon National University 255, Jungang‐ro, Suncheon‐si Jellanam‐do 57922 Republic of Korea

**Keywords:** biomedical, nanomaterial, stimulus responsive

## Abstract

Stimuli‐responsive materials (SRMs) are materials that change properties when exposed to external or internal stimuli. They respond to physiological changes within cells and tissues, as well as external triggers including light, magnetic fields, ultrasound, and electricity. In medicine, SRMs have diverse applications spanning drug delivery, tissue engineering, and diagnostics. They enable targeted drug release at specific times and locations, facilitate tissue generation and repair, and enhance disease detection capabilities. Beyond medical uses, SRMs are employed in smart coatings and artificial muscle systems. The breadth of biomedical applications for SRMs is extensive, generating substantial research into novel and innovative material development. Challenges in creating safe and efficient SRMs for medical treatments have driven innovative approaches in two key areas: functionalizing and modifying naturally occurring materials and developing new synthetic nanomaterials. The complexity of producing effective SRMs has necessitated creative solutions to overcome safety and efficiency barriers in medical applications. This ongoing research continues to expand the potential therapeutic uses of these responsive materials. This review examines literature focused on SRM development for external stimuli responses, particularly light, magnetic fields, ultrasound, and electricity, rather than covering the complete spectrum of stimuli‐responsive applications.

## Graphical abstract

Stimulus‐responsive materials (SRMs) hold great promise for use in a wide range of biomedical applications. This review covers four stimulus modalities, namely, electrical, optical, magnetic, and ultrasound, and their associated SRMs. It provides a summary of the materials in each modality, their development, and current research perspectives. This review also provides a detailed analysis of the key challenges for translating these SRM technologies into clinical use.

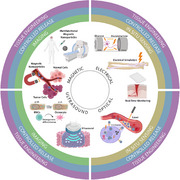

## 1 Introduction

**Figure 1:**
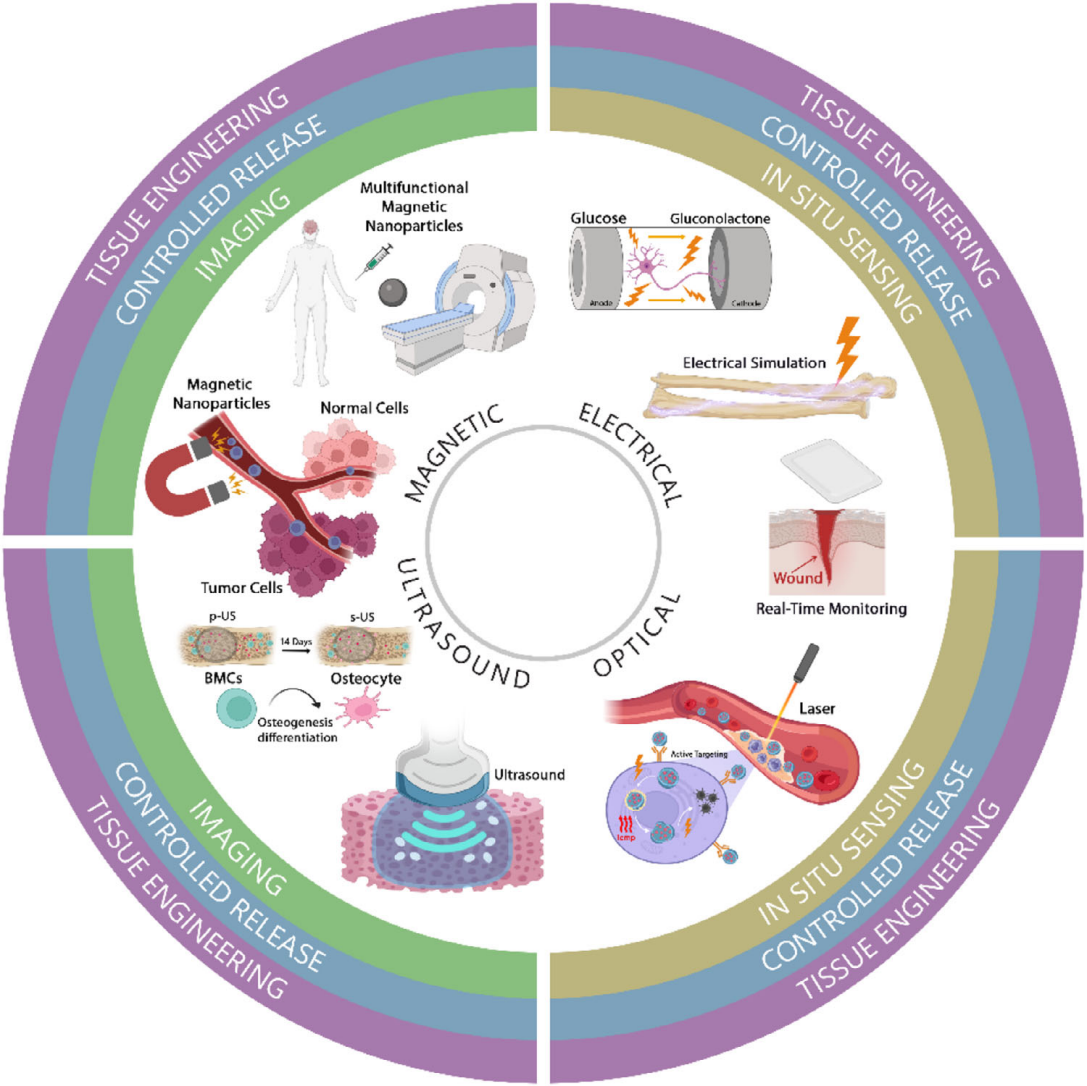
Schematic illustration of stimuli‐responsive materials in which the stimuli modalities are aligned with biomedical applications.

Stimulus‐responsive materials (SRMs) represent a cutting‐edge class of materials whose properties change in response to external or internal stimuli. These dynamic materials can respond to physiological changes within cells or tissues, as well as to external triggers such as light, magnetic fields, ultrasound, or electricity. In the medical field, SRMs have revolutionized numerous applications ranging from drug delivery systems, imaging, tissue engineering, and diagnostics (**Figure** **1**). The versatility of these materials allows for precise control over when and where drugs are released; how tissues are regenerated, repaired, and imaged; and how diseases are detected with improved sensitivity and specificity.

The development of novel and innovative SRMs has been driven by the challenges associated with creating safe and effective materials for treating medical conditions. This has led to sophisticated approaches for functionalizing and modifying naturally occurring materials, as wellas the creation of new synthetic nanomaterials with unique responsive properties. Research in this field is rapidly evolving, with studies focusing on optimizing these materials for specific applications while ensuring their safety and efficacy in medical contexts. SRMs have been fabricated from a diverse array of materials, with the choice of materials largely dependent on the intended external stimulus. For light‐responsive applications, gold nanoparticles (AuNPs) have been extensively investigated because of their unique optical properties.^[^ ^1^, ^2^, ^3^ ^]^ When magnetic fields are the stimulus source, superparamagnetic materials such as iron oxide (Fe_3_O_4_) are commonly employed because of their ability to respond to and be manipulated by external magnetic fields.^[^ ^4^, ^5^, ^6^ ^]^ For ultrasound stimuli, an extensive body of work has been done on a range of materials, including lipids,^[^ ^7^, ^8^ ^]^ polymers,^[^ ^9^, ^10^, ^11^ ^]^ and metals.^[^ ^12^, ^13^ ^]^ These materials can undergo structural changes or release encapsulated substances when exposed to ultrasound waves. Electrically responsive materials such as organic conducting polymers (OCPs),^[^ ^14^, ^15^ ^]^ carbon‐based nanomaterials (graphene^[^ ^16^, ^17^, ^18^ ^]^ and carbon nanotubes^[^ ^19^, ^20^ ^]^) have been developed to harness the beneficial effects of this stimulus source.

These SRMs have been applied to a wide range of biomedical applications ranging from drug delivery, tissue engineering, sensing, and imaging. SRMs have transformed drug delivery by enabling targeted, controlled release of therapeutic agents. Controlled release of drugs has been demonstrated through the use of these stimulus modalities,^[^ ^21^, ^22^, ^23^, ^24^ with some modalities facilitating the delivery of therapeutics to challenging locations, such as across the blood–brain barrier.^[^ ^25^, ^26^, ^27^ ^]^ In tissue engineering, SRMs provide dynamic scaffolds that can mimic the native extracellular matrix while offering additional functionalities. Electrical stimulation through conductive materials has shown significant promise in nerve regeneration^[^ ^15^, ^28^ ^]^ by providing a supportive environment for nerve cells and promoting their growth and differentiation. Similarly, cardiac tissue engineering benefits from electrically conductive scaffolds that support synchronized contractions of engineered heart tissue.^[^ ^29^ ^]^ SRMs have enhanced diagnostic capabilities through improved imaging contrast agents and biosensors. Magnetic responsive nanoparticles serve as contrast agents in magnetic resonance imaging (MRI) and as tracers in the emerging field of magnetic particle imaging (MPI).^[^ ^30^ ^]^ Ultrasound contrast agents, typically in the form of microbubbles, enhance the visualization of blood vessels and organs.^[^ ^31^, ^32^, ^33^, ^34^ ^]^

The selection of the most suitable stimulus for a specific biomedical application requires careful consideration of various factors. These include tissue penetration, spatial resolution, temporal control, safety considerations and equipment requirements, with the practical implementation of different stimuli of varying complexity. This, in turn, requires considerable effort to produce nanomaterials that respond appropriately to these stimuli to invoke the desired biomedical outcome. As these challenges are addressed and new directions pursued, stimuli‐responsive materials hold tremendous potential to revolutionize healthcare by enabling more precise, personalized, and effective medical interventions. The interdisciplinary nature of this field, which combines expertise in materials science, engineering, biology, and medicine, will continue to drive innovation and advancement in biomedical applications.

This review provides an overview of current advancements in stimuli‐responsive materials that have applications in the biomedical area, discussing fundamental principles, recent innovations, and future directions in research. Where appropriate, the reader is directed throughout the article to other valuable reviews that provide a deeper discussion in focused areas of SRM. It focuses on four stimulus modalities, namely, electrical, optical, magnetic, and ultrasonic, and details the fundamentals of materials suited for each modality. In addition, a summary framework is provided to demonstrate what factors should be considered when choosing the most appropriate stimulus modality and material to suit biomedical applications. The approach taken in this review is to assess a wide range of SRMs and stimulation modalities in the context of biomedical applications. A novel section of this review is the final detailed assessment of the technology readiness of these SRM technologies and discussions of the challenges these face for health sector translation.

## 2 Electrical Responsiveness

Having established above the broad landscape of stimuli‐responsive materials and their transformative potential in biomedical applications, we now turn our attention to one of the most direct and controllable stimulus modalities. Electrical stimuli offer unique advantages in biomedical contexts because of their ability to interface directly with the body's own tissue and regenerative systems. This natural compatibility makes electrically responsive materials particularly well‐suited for applications requiring precise temporal control and direct cellular interaction.

Electroactive materials have had a significant impact on biomedical applications by providing unique capabilities for sensing, actuation, and controlled drug delivery within biological environments. These smart materials can change their properties in response to electrical stimuli, enabling precise control over cellular interactions, tissue regeneration, and therapeutic delivery. Their ability to mimic the electrical signaling of cells and natural tissues makes them particularly valuable for neural interfaces and cardiac applications, whereas their tunability allows for dynamic scaffolds that can promote healing in response to physiological conditions.

### 2.1 Fundamental Principles

Electrically responsive materials, also known as electroactive materials, present a change in their physical or chemical properties in response to an applied electric field or current. This change can manifest as a change in shape, volume, conductivity, optical properties, or other characteristics. The mechanisms of electrical responsiveness include charge transfer, electrochemical reactions, electrophoretic effects, and dielectric polarization.^[^ ^35^ ^]^

Several mechanisms influence the ways in which materials respond to electrical stimuli. Charge transfer describes the movement of electrons or ions within a material in response to an electric field. This fundamental process controls how materials conduct or interact with electrical charges and is crucial for the function of conductive polymers and many nanomaterials, such as carbon nanotubes and graphene.^[^ ^36^ ^]^ Electrochemical reactions, including redox reactions (oxidation and reduction), can be induced by applying an electrical potential. These reactions lead to chemical changes within materials, altering their properties, as observed in certain hydrogels.^[^ ^37^ ^]^ Furthermore, electrophoretic effects describe the movement of charged particles, such as those within a hydrogel, under the influence of an electric field. This phenomenon enables control over processes such as drug release and molecular movement.^[^ ^38^ ^]^ Last, dielectric polarization describes how materials respond to electric fields by aligning their internal charges, specifically the molecular dipoles. This alignment can significantly influence material properties, especially in liquid crystals. These diverse mechanisms allow for a wide range of material responses to electrical stimuli, enabling their use in various biomedical applications.^[^ ^39^ ^]^

### 2.2 Classification of Electrically Responsive Materials

**Figure 2:**
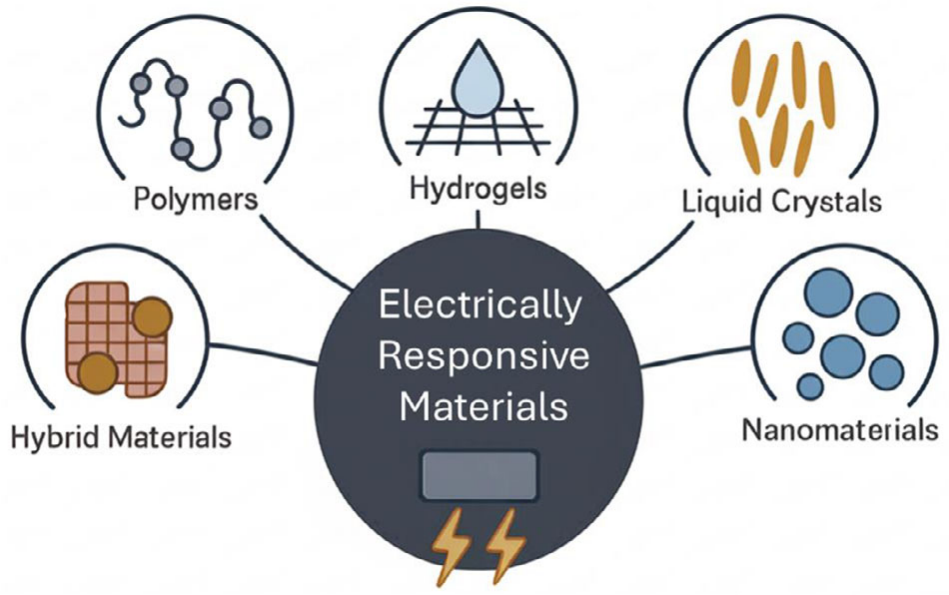
Schematic overview of the major classes of electrically responsive materials for biomedical applications.

Understanding the diverse categories of electrically responsive materials is crucial for tailoring material properties to specific biomedical applications.^[^ ^40^ ^]^ These materials can be broadly classified on the basis of their composition and response mechanisms. **Figure** **2** provides a schematic overview of the primary classes: conductive polymers, hydrogels, liquid crystals, nanomaterials, and hybrid materials. Each presents unique characteristics that make them suitable for applications ranging from tissue engineering to drug delivery and biosensing.

The subsequent subsections present the fundamental principles, characteristic properties, and specific biomedical applications unique to each of these material classifications.

#### 2.2.1 Conductive Polymers

Conductive polymers (CPs) are organic polymers that can conduct electricity, unlike most plastics, which are insulators. CPs are a unique class of materials that combine the electrical conductivity of metals with the processability and flexibility of plastics. Their ability to conduct electricity stems from their conjugated backbone, which consists of alternating single and double bonds that create a delocalized “sea” of electrons. These delocalized electrons can move freely along the polymer chain, enabling efficient charge transport.^[^ ^41^ ^]^ Furthermore, the processability of CPs allows their fabrication into various forms, such as films, fibers, and coatings, making them attractive for a wide range of applications, including biomedicine.^[^ ^42^ ^]^

**Figure 3:**
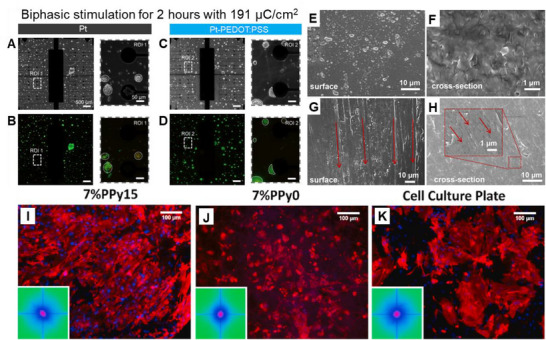
Conductive polymer examples in biomedical applications. A–D) PEDOT:PSS‐coated platinum electrodes for neural stimulation (Reproduced with permission^[^ ^43^ ^]^ Copyright 2023, ACS) presenting (A,B) microscopy images showing the Pt and Pt‐PEDOT:PSS electrodes before stimulation and (C,D) microscopy images showing the Pt and Pt‐PEDOT:PSS electrodes after 2 h of biphasic stimulation (191 µC.cm^−2^). The green fluorescence indicates the neuronal response (e.g., growth or activity). These images demonstrate the enhanced neuronal response on the PEDOT:PSS‐coated electrodes, highlighting their potential for neural stimulation applications. E–H) Microstructural characterization of a PANI hydrogel with self‐reinforcing behavior. Reproduced with permission^[^ ^46^ ^]^ Copyright 2023, ACS using SEM imaging to visualize E) the original hydrogel surface, revealing an isotropic morphology; F) the original hydrogel cross‐section, providing insight into the initial internal structure; G) the hydrogel surface after mechanical stretching, showing the development of aligned structural features; and H) the hydrogel cross‐section after mechanical stretching, with a magnified inset highlighting the aligned microstructure. These images illustrate how the microstructure of the hydrogel is altered by stretching, contributing to its improved mechanical performance. I–K) PPy‐encapsulated silk fibroin fibers for cardiac tissue engineering. Reproduced with permission^[^ ^45^ ^]^ Copyright 2021, Elsevier. Fluorescence microscopy images of cardiomyocytes cultured on 7% PPy15 (I), 7% Ppy0 (J), and a cell culture plate (K). Red and green fluorescence indicate different cell types or markers. These images show the improved cell growth and function of the PPy‐encapsulated fibers, demonstrating their potential for cardiac tissue engineering.

A number of conductive polymers have been extensively investigated for their use in biomedical applications, with some of the most promising candidates discussed in further detail here. Poly(3,4‐ethylenedioxythiophene) (PEDOT) is renowned for its excellent electrical conductivity, biocompatibility, and stability in physiological environments. It is often preferred for biomedical applications because of these characteristics. PEDOT is frequently used in its PEDOT:PSS form, where PSS (polystyrene sulfonate) acts as a counterion and improves the processability and flexibility of the material. Consequently, PEDOT and PEDOT:PSS have found applications in neural interfaces, tissue engineering, and biosensors.^[^ ^43^ ^]^ In contrast, polyaniline (PANI) is notable for its ease of synthesis, making it a cost‐effective option. Its conductivity can be tuned over a wide range by controlling the doping level (introducing impurities that affect the number of charge carriers). This tunable conductivity makes PANI versatile for applications ranging from sensors to actuators.^[^ ^44^ ^]^ However, the biocompatibility of PANI is highly variable and depends on its formulation and processing, whereas in common formulations, PEDOT:PSS often has good biocompatibility. Finally, in addition to its good electrical conductivity and biocompatibility, polypyrrole (PPy) has the unique ability to undergo reversible oxidation and reduction reactions, making it particularly attractive for applications where dynamic control over its electrical and chemical properties is desired. This redox activity, coupled with its versatile processability, has led to its use in various biomedical applications, including drug delivery systems that release therapeutic agents in response to electrical signals and bioactuators that mimic the mechanical movements of biological tissues.^[^ ^45^ ^]^ **Figure** **3** presents promising applications of conductive polymers in biomedicine while highlighting key areas for further investigation.

PEDOT:PSS, as shown in Figure 3A–D, improves the stability of neural stimulation electrodes by preventing platinum dissolution, a critical factor for long‐term implantable devices. However, the exact mechanism of this protection and the long‐term stability of the PEDOT:PSS coating under various stimulation conditions require further research. Figure 3E–H presents a comprehensive microstructural characterization of the self‐reinforcing behavior of the PANI hydrogel via SEM imaging. This technique clearly shows a correlation between mechanical stretching and microstructural transformation, with the hydrogel initially displaying an isotropic surface morphology and a distinct initial internal arrangement in its cross‐section. Mechanical stretching, however, triggers a significant alteration in the microstructure, leading to the emergence of aligned structural features in both surface and cross‐sectional views. This stretching‐induced microstructural transformation involves the reorientation and alignment of randomly distributed PVA segments and the enhanced formation of intermolecular hydrogen bonds between PVA chains, which directly explains the observed improvement in the mechanical performance of the material.^[^ ^47^ ^]^ In Figure 3I–K, Ppy‐encapsulated fibers were shown to support cardiomyocyte growth and alignment, indicating promise for cardiac tissue engineering. However, optimizing material properties, such as the PPy concentration, is crucial to achieve an optimal cell response and functional tissue formation. These findings highlight the versatility and significant potential of conductive polymers in addressing critical needs in tissue engineering, drug delivery, and biosensing. While challenges remain in fully understanding their long‐term stability, optimizing their properties for specific applications, and integrating them into various biomedical devices, the future of conductive polymers in biomedicine holds great potential for advancing healthcare.^[^ ^45^ ^]^

#### 2.2.2 Hydrogels

Hydrogels are crosslinked polymer networks that present high water content within their network structure, giving them a consistency similar to that of biological tissues. Composite hydrogels are discussed in several sections of the review because they are applicable across several SRM technologies and stimuli modalities. A recent review by Huang et al.^[^ ^48^ ^]^ provides a comprehensive analysis of nanocomposite hydrogels across a range of biomedical applications and provides valuable additional information for this review. The biocompatibility of hydrogels, along with their tunable mechanical properties and responsiveness to various stimuli, including electrical signals, make them attractive for biomedical applications. They can also encapsulate drugs and cells, which further enhances their versatility.^[^ ^49^ ^]^ Electrically responsive hydrogels (ERHs) are a subclass of hydrogels that respond to electric fields by altering their swelling, mechanical properties, or drug release. ERHs have potential for various biomedical applications because of their precise controllability, which is attributed to several mechanisms, including Joule heating, electrophoretic effects, electrochemical reactions, and changes in polymer chain conformation.^[^ ^50^ ^]^

**Figure 4:**
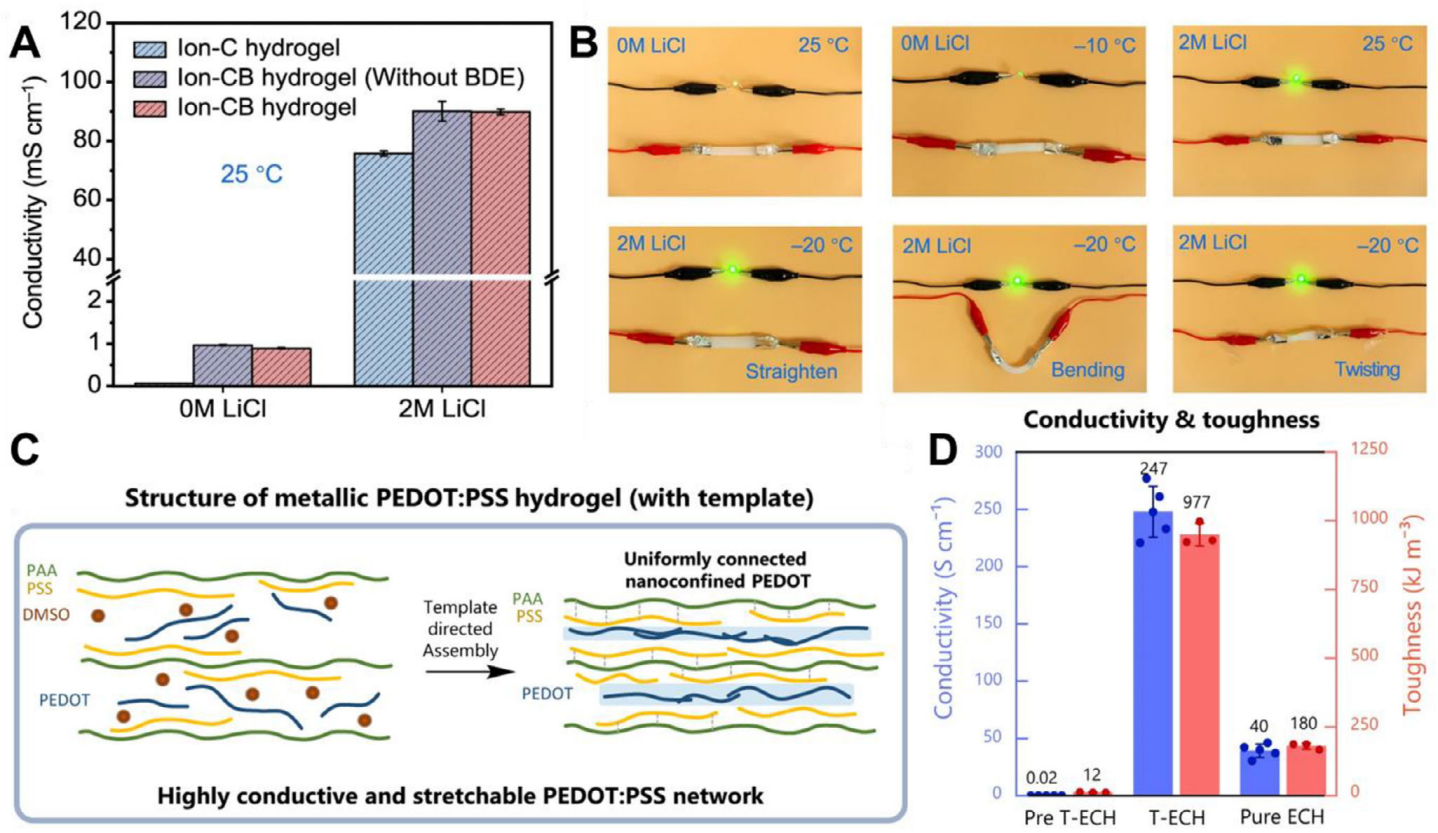
The generation of ERHs with enhanced conductive properties through different design strategies. A) Comparison of the ionic conductivity of cellulose‐based hydrogels at 25 °C, with the ionically conductive cellulose‐bentonite (ion‐CB) hydrogel (2 m LiCl) showing significantly greater conductivity (89.9 mS cm^−1^) than the control formulation (0 m LiCl). B) Visual demonstration of the Ion‐CB hydrogel's practical ionic conductivity and flexibility; the hydrogel powers an LED even when straightened, bent, twisted, or at various temperatures (e.g., −20 °C). C) Schematic of template‐directed assembly, where a polyacrylic acid (PAA) template guides the formation of a uniformly connected, nanoconfined PEDOT network, creating a highly conductive and stretchable PEDOT:PSS hydrogel (T‐ECH). C) Comparison of the electrical conductivity and toughness of the PEDOT:PSS‐based hydrogels, illustrating that the template‐assembled T‐ECH (center bars) achieves significantly higher conductivity (247 S cm^−1^) than does its precursor (Pre T‐ECH) or a nontemplated version (Pure ECH). A,B) Reproduced with permission.^[^ ^54^ ^]^ Copyright 2022, Nature. C,D) Reproduced with permission.^[^ ^55^ ^]^ Copyright 2023, Nature.

One common type is conductive polymer‐based hydrogels, which incorporate conductive polymers, such as PEDOT, PANI, or PPy, within the hydrogel matrix. Electrical stimulation can affect the conformation, oxidation state, or interactions of the polymer within the hydrogel network, which in turn alters the overall properties of the hydrogel.^[^ ^51^ ^]^ Another type is nanoparticle‐based hydrogels, in which conductive nanoparticles such as gold, carbon nanotubes, or graphene are embedded within the hydrogel. The nanoparticles can respond to an electric field through mechanisms such as Joule heating (generating heat) or by influencing the crosslinking density, which affects the overall properties of the hydrogel.^[^ ^52^ ^]^ Other ERHs might include those incorporating electroactive polymers that respond directly to electric fields or those that respond to changes in the ion concentration induced by an electric field. These mechanisms are often more complex and specific to different applications.^[^ ^53^ ^]^ Illustrating these principles, recent advancements in electrically responsive hydrogels have led to materials with significant ionic conductivity, which are often developed via nanocomposite designs, and others with high electronic conductivity, which are frequently achieved through strategies such as template‐directed assembly of conductive polymer networks, as presented in **Figure** **4**.

Figure 4 reported that nanocomposite hydrogels incorporating cellulose and bentonite (ion‐CB hydrogels) doped with 2 m LiCl demonstrated impressive ionic conductivity, reaching ≈89.9 mS cm^−1^ at 25 °C. This formulation significantly outperforms formulations without bentonite or with lower salt concentrations (Figure 4A). The practical utility of this high ionic conductivity, combined with the material's mechanical flexibility and freezing tolerance, is highlighted by the ability of these Ion‐CB hydrogels to power a light‐emitting diode (LED). This functionality is maintained even when the hydrogel is subjected to physical deformation, such as bending and twisting, or at temperatures as low as −20 °C (Figure 4B). The enhanced ion transport in such systems is attributed to the nanoconfined, intercalated structure formed by cellulose‒bentonite interactions, which can serve as fast ion transport channels.^[^ ^54^ ^]^

In the development of electronically conductive hydrogels, template‐directed assembly has emerged as a powerful strategy for creating highly efficient conductive networks. By employing a polyacrylic acid (PAA) template, a uniformly connected and nanoconfined PEDOT network can be precisely engineered within a stretchable hydrogel matrix (Figure 4C). This method effectively mitigates the formation of large, insulating PSS aggregates and reduces the degree of conformational disorder that can limit charge transport in conventionally prepared PEDOT:PSS hydrogels. Consequently, the template‐assembled conductive hydrogel (T‐ECH) exhibited markedly superior electrical conductivity, achieving values as high as 247 S cm^−1^. This represents a significant improvement over precursor materials or pure PEDOT:PSS hydrogels, while the material also retains good mechanical toughness (Figure 4D).^[^ ^55^ ^]^ Overall, these demonstrations of high ionic and electronic conductivity in different hydrogel systems highlight the versatility of these materials, leading the way for their broader implementation in advanced bioelectronic devices and electrically mediated therapeutic strategies.^[^ ^56^ ^]^

#### 2.2.3 Liquid Crystals

Liquid crystals (LCs) are a unique phase of matter that exhibit properties of both liquids (fluidity) and solid crystals (order). This combination gives them distinct optical and electrical properties. LCs are categorized by their molecular order: nematic (simple alignment), smectic (layered), and cholesteric (helical). Nematic LCs are most relevant for electrically responsive systems because they reorient in electric fields, changing their optical properties. This approach is useful for applications such as biosensors, microfluidic devices, and adaptive optics.^[^ ^57^ ^]^ The electrical responsiveness of liquid crystals stems from the interaction between electric fields and the dipole moments of the LC molecules. Each LC molecule has a separation of positive and negative charges, creating a dipole moment. When an electric field is applied, these dipoles experience a torque, causing the molecules to align with the field. This reorientation affects the optical properties of the LC, particularly its birefringence, which is the difference in the refractive indices of light polarized in different directions. This change in birefringence can be detected and used for sensing applications or to modulate light transmission in displays and other optical devices.^[^ ^58^ ^]^

Some notable examples of electrically responsive liquid crystals with promising biomedical applications include 5CB (4‐cyano‐4′‐pentylbiphenyl), a common nematic liquid crystal that is widely used because of its high birefringence and rapid response to electric fields. This responsiveness makes it suitable for applications such as biosensors, where changes in molecular alignment can be used to detect the presence of specific biomolecules.^[^ ^59^ ^]^ For example, 5CB‐based sensors have been developed for detecting DNA hybridization and the presence of viruses. MBBA (N‐(4‐methoxybenzylidene)‐4‐butylaniline) is another nematic LC that strongly responds to electric fields, in addition to its temperature sensitivity. Its electrical responsiveness has been explored for use in microfluidic devices, where electric fields can control the orientation of MBBA and manipulate the flow of fluids in microchannels. Furthermore, ferroelectric liquid crystals, which possess spontaneous polarization, exhibit a very fast switching response to electric fields. This makes them attractive for applications such as high‐speed displays and spatial light modulators, which can be used for advanced imaging techniques. These examples illustrate the diverse range of electrically responsive LCs and their potential to enable novel biomedical technologies.^[^ ^60^ ^]^

#### 2.2.4 Nanomaterials

Nanomaterials are a class of materials that present at least one external dimension at the nanoscale, typically defined as 1 to 100 nanometers. This small size gives them unique properties, including a high surface area‐to‐volume ratio, quantum effects leading to novel optical and electrical behaviors, and often enhanced electrical conductivity and mechanical strength compared with those of bulk materials. These characteristics make them attractive for biomedical applications such as targeted drug delivery, enhanced imaging, and tissue engineering, as they can interact with cells and biomolecules at the nanoscale.^[^ ^61^ ^]^

Electric fields can influence the emission characteristics of nearby fluorescent probes by altering the emission intensity, peak emission wavelength, and fluorescence lifetime, making them valuable tools for sensing applications. The most widely used probes in this area are molecular voltage sensors. These sensors rely on changes in molecular orientation, position, or electronic structure to detect variations in electric fields, as detailed in the review by.^[^ ^62^ ^]^ In recent years, the development of inorganic nanomaterials for electric field sensing has gained traction because of their high photostability, tunable emission properties, and nanoscale sensitivity. These materials operate primarily through the quantum‐confined Stark effect, where exposure to an electric field induces a shift in the peak emission wavelength due to the redistribution of charge carriers in confined nanostructures. Among the most extensively studied materials in this category are quantum dots (QDs). Experiments by Caglar et al.^[^ ^63^ ^]^ demonstrated the use of CdSe/CdS and InP/ZnS quantum dots for monitoring membrane potential shifts in live *Xenopus laevis* retinal ganglion cell axons. Their study revealed significant changes in the quantum dot emission intensity (up to 66%), indicating strong sensitivity to fluctuating electric fields. Further work by Park et al.^[^ ^64^ ^]^ explored the functionalization of CdSe/CdS nanorods with membrane‐inserting protein structures, which enabled the direct integration of nanorods into cellular membranes. Their results demonstrated single‐particle sensitivity to voltage fluctuations, highlighting the potential of quantum dot‐based systems for nanoscale electrophysiological sensing.

**Figure 5:**
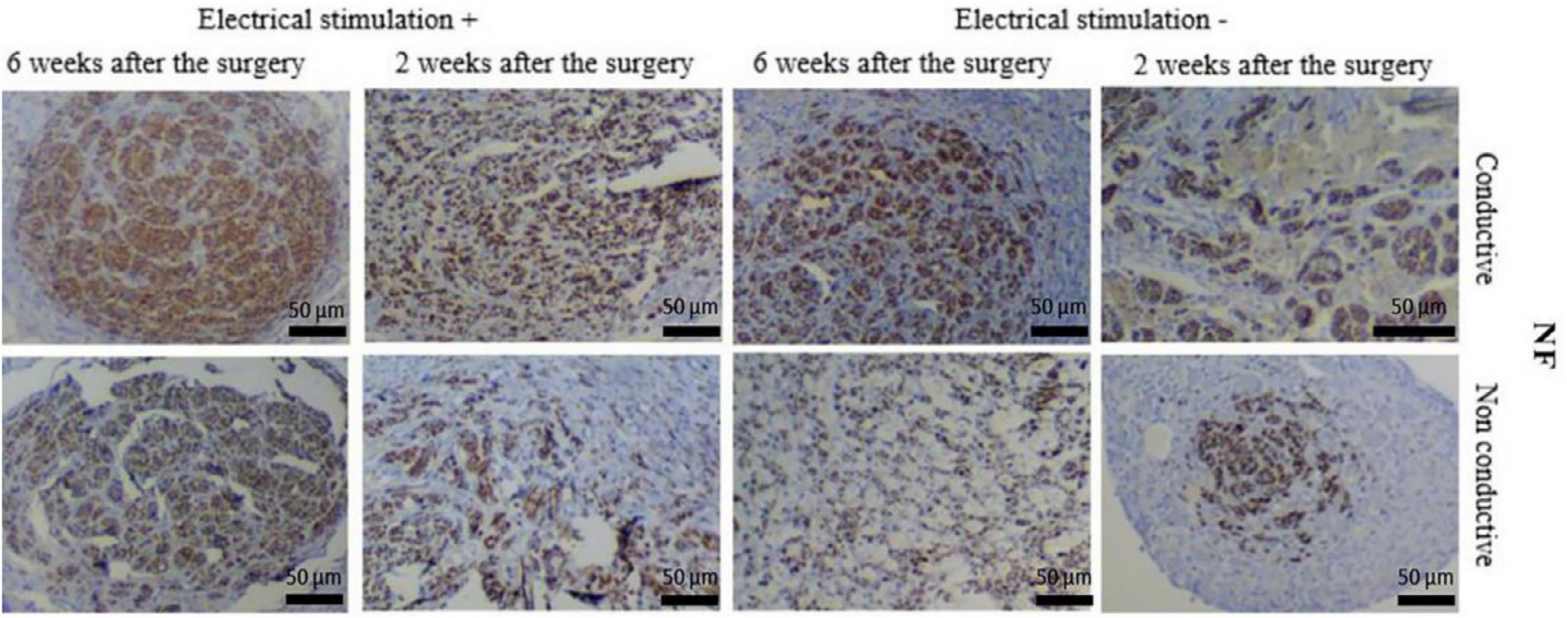
Immunohistochemical analysis of nerve regeneration within conductive and nonconductive scaffolds, with (+) and without (−) electrical stimulation (ES), at 2 and 6 weeks postsurgery. Cross‐sections are stained for neurofilaments (NFs, brown) to indicate the presence of axons. Compared with the other conditions, the conductive scaffold combined with ES results in a qualitatively greater presence and organization of neurofilaments at 6 weeks, suggesting enhanced axonal regeneration. Scale bars = 50 µm. Reproduced with permission.^[^ ^68^ ^]^ Copyright 2024, Nature.

Carbon nanotubes (CNTs) are cylindrical nanostructures formed from rolled‐up sheets of graphene. They exhibit exceptional electrical conductivity, high mechanical strength, and a large surface area. Additionally, their surfaces can be readily functionalized with various molecules, allowing tailored interactions with biological systems. These properties make CNTs attractive for applications such as neural interfaces, where they can facilitate electrical communication with neurons, and in tissue engineering, where they can provide structural support and electrical cues to guide cell growth. Graphene is also highly flexible and transparent, making it suitable for flexible electronics and biosensors. For example, graphene‐based biosensors have shown promise in detecting minute changes in electrical signals associated with specific biomolecules, such as neurotransmitters or DNA.^[^ ^65^ ^]^ Metallic nanoparticles, such as those made of gold or platinum, exhibit unique electrical properties that make them responsive to electrical stimulation. For example, gold nanoparticles can be used to increase the electrical conductivity of hydrogels or other biomaterials, increasing their responsiveness to electrical fields. This approach can be used to control the release of drugs from hydrogels or to stimulate cells in tissue engineering applications. On the other hand, platinum nanoparticles can catalyze electrochemical reactions in response to electrical stimulation, which can be used for applications such as biosensing and energy conversion.^[^ ^66^ ^]^ Quantum dots (QDs), with their size‐dependent electronic properties, can be used to create electrically responsive sensors and actuators. QDs can be incorporated into field‐effect transistors (FETs) to create electrically stimulated devices for biomedical applications. By measuring changes in the electrical conductivity of the QDs in response to an electric field, these FET‐based devices can be used to stimulate cells or tissues, offering potential for therapies related to nerve regeneration, wound healing, and muscle stimulation.^[^ ^67^ ^]^ The therapeutic potential of electrically stimulated nanomaterial‐based systems in promoting tissue regeneration, particularly in nervous tissue, is further evidenced by histological studies. For example, the application of electrical stimulation in conjunction with conductive nanomaterial‐based scaffolds has been shown to significantly influence the presence and organization of axons in regenerated nerve tissue, as illustrated by neurofilament staining of sciatic nerve cross‐sections **Figure** **5**.

The histological evidence presented in Figure 5 highlights the beneficial role of nanomaterial‐based conductive scaffolds when combined with ES for promoting axonal regeneration. Neurofilament (NF) staining, which indicates the presence of axons, is more abundant and appears more organized in the group that received a conductive scaffold (silk fibroin with gold nanoparticles) under electrical stimulation, particularly at the 6‐week time point (Figure 5, top row, “Electrical stimulation +”, 6 weeks after surgery). This enhanced axonal presence suggests that the conductivity imparted by the gold nanoparticles facilitates more effective delivery or influence of the electrical cues to the regenerating nerve tissue. On the other hand, the nonconductive scaffolds, especially those without electrical stimulation (Figure 4, bottom row, “Electrical stimulation ‐”), presented a sparser neurofilament presence, indicative of less successful axonal regrowth. These visual comparisons emphasize the synergistic advantage of combining nanomaterials to create conductive microenvironments with electrical stimulation therapies for neural tissue engineering, aligning with strategies to improve outcomes in peripheral nerve repair.^[^ ^68^ ^]^

**Figure 6:**
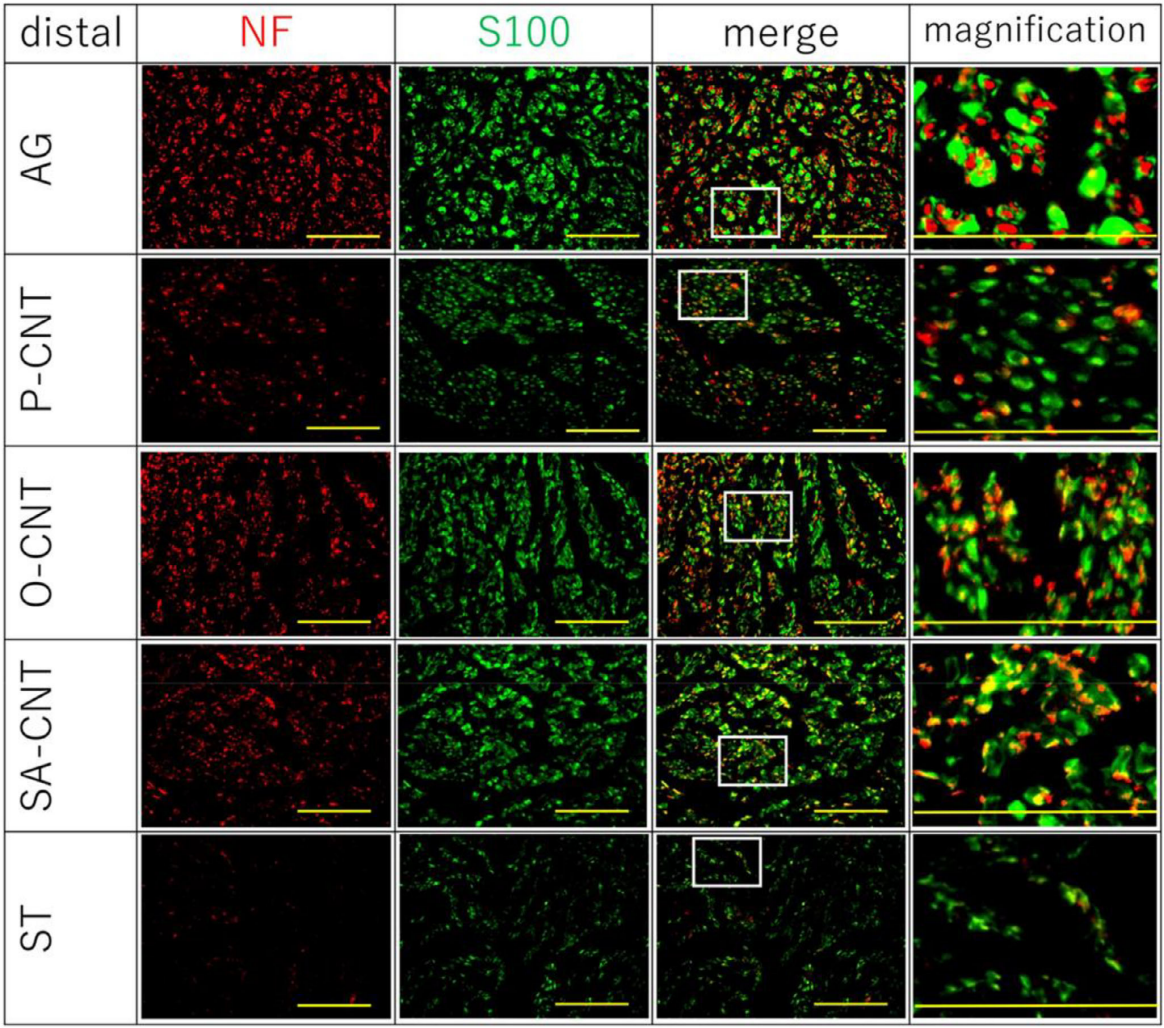
Assessment of axonal regeneration and the presence of Schwann cells in the distal nerve segment 8 weeks postimplantation using different carbon nanotube (CNT) yarn scaffolds. Immunofluorescence staining revealed neurofilaments (NFs, red), indicating axons, and S100 (green), indicating Schwann cells. The groups included autografts (AGs), pristine CNTs (P‐CNTs), ozone‐treated CNTs (O‐CNTs), strongly acid‐treated CNTs (SA‐CNTs), and silicon tube (ST) controls. The oxidized CNT groups (O‐CNT and SA‐CNT) show notable axonal presence and Schwann cell alignment, indicating their potential to support nerve regeneration. Scale bars are presented in each micrograph. Reproduced with permission^[^ ^69^ ^]^ Copyright 2023, Nature.

CNT‐based scaffolds have also been extensively investigated to determine the effects of the nanomaterial composition and surface properties on neural regeneration. Modifications to CNT surfaces, such as oxidation, can influence cellular interactions and regenerative outcomes, as shown by the differing densities of axons and Schwann cells in regenerated nerve tissue **Figure** **6**.

The immunohistochemical analysis presented in Figure 6 further highlights the potential of CNT‐based scaffolds in peripheral nerve regeneration and highlights the importance of their surface properties. The figure illustrates the presence of neurofilaments (NFs, marked with axons) and S100‐positive Schwann cells in the distal nerve segment 8 weeks after the implantation of various CNT yarn constructs. Notably, strongly acid‐treated CNTs (SA‐CNT) support a robust regenerative response characterized by significant axonal presence and accompanying Schwann cells, with outcomes comparable to those of the autograft (AG) group and superior to those of pristine CNTs (P‐CNT). Compared with untreated CNTs, ozone‐treated CNTs (O‐CNTs) also produced favorable results. In contrast, the empty silicon tube (ST) control group presented minimal signs of regeneration. These findings suggest that surface modifications of CNTs, such as oxidation, play crucial roles in enhancing their biocompatibility and capacity to promote essential regenerative processes such as neurite outgrowth and Schwann cell migration.^[^ ^69^ ^]^

#### 2.2.5 Hybrid Materials and Composites

Hybrid materials and composites offer a powerful strategy for tailoring material properties and achieving enhanced functionality. These materials are formed by combining two or more distinct material types, resulting in a synergistic combination that overcomes the limitations of the individual components. For example, conductive polymers exhibit excellent electrical conductivity but may lack optimal biocompatibility or mechanical properties for certain biomedical applications. On the other hand, hydrogels offer biocompatibility and tunable mechanical properties but typically lack electrical conductivity. By combining a conductive polymer with a hydrogel, researchers can create a hybrid material that presents both electrical conductivity and biocompatibility, expanding its potential applications in areas such as tissue engineering and drug delivery. Similarly, combining conductive polymers with nanomaterials can lead to improved electrical, mechanical, or optical properties or enhanced stability compared with the use of either component alone.^[^ ^50^ ^]^

Combining conductive polymers with hydrogels creates a material that is both electrically conductive and biocompatible. This type of composite is attractive for tissue engineering, where the conductive polymer can provide electrical cues to guide cell growth, and for drug delivery, where electrical signals can trigger drug release. The incorporation of conductive nanoparticles into hydrogels can also enhance their electrical properties and responsiveness to stimuli, making them suitable for tissue engineering and biosensing applications.^[^ ^70^ ^]^ Moreover, other hybrid materials relevant to electrically responsive biomedical systems include those incorporating liquid crystals or piezoelectric materials. These materials offer unique capabilities, such as the ability to respond to both electrical and optical stimuli or generate electrical signals in response to mechanical stress, opening up possibilities for new types of sensors, actuators, and energy generation devices.^[^ ^47^ ^]^

### 2.3 Applications of Electrically Responsive Materials

Building on the previous discussion of electrically responsive materials, this section explores their key applications in biomedical engineering. The focus will be on how the electrical responsiveness of these materials is being used to address challenges in tissue engineering, drug delivery, and biosensing.

#### 2.3.1 Tissue Engineering

Electrical stimulation can play a crucial role in tissue engineering, as it influences cellular behavior and promotes tissue regeneration by modulating the cell membrane potential, activating signaling pathways, and controlling protein synthesis. These effects guide key processes such as cell proliferation, differentiation, and organization, enabling the development of electrically responsive materials for scaffolds and devices in various tissue engineering applications.^[^ ^71^ ^]^

**Figure 7:**
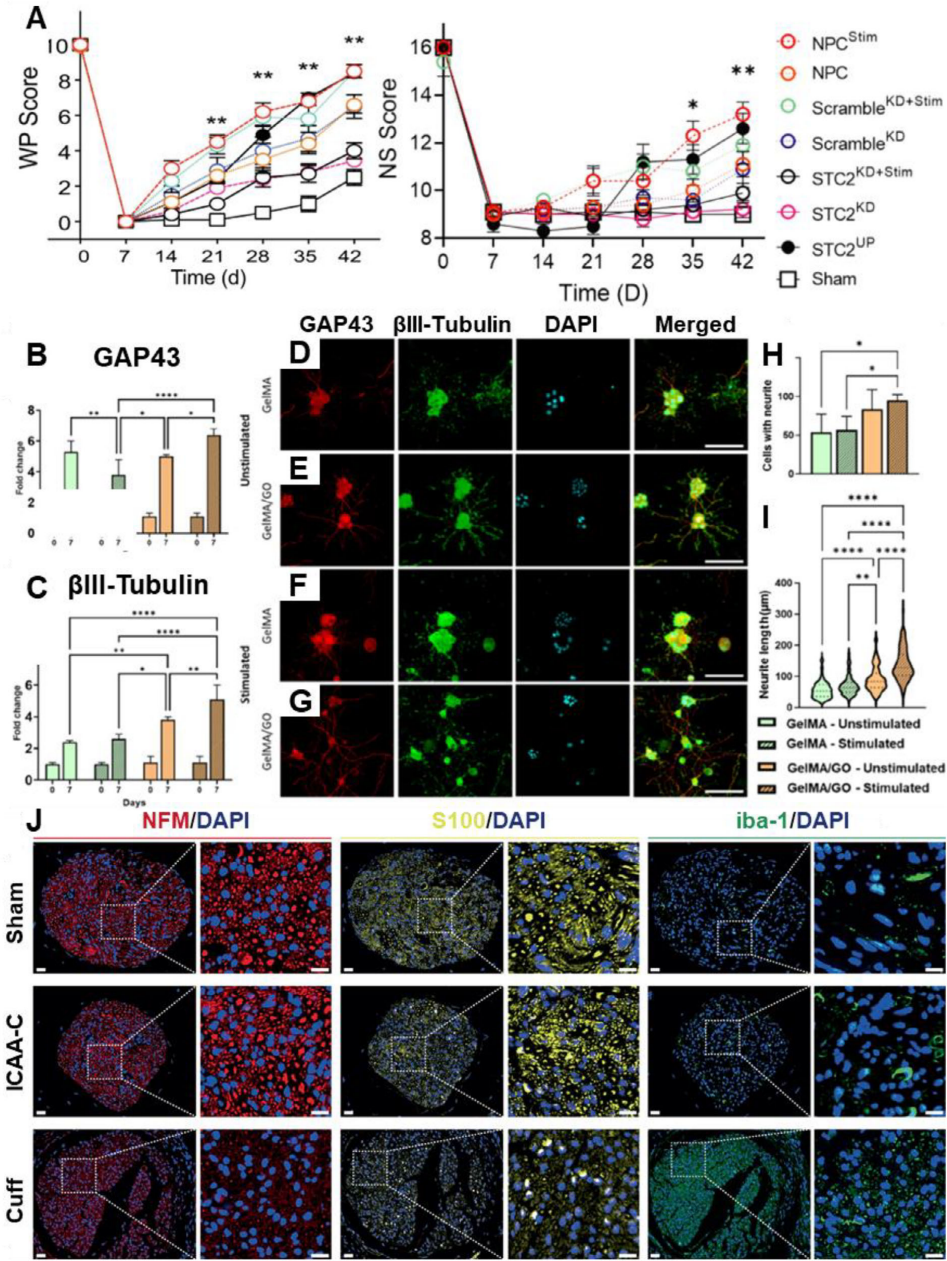
Conductive polymers and electrical stimulation for nerve regeneration. A) Electrical modulation of transplanted stem cells in a rodent model of stroke, presenting a schematic of the experimental setup showing the transplantation of stem cells into the stroke lesion site and the application of electrical stimulation via conductive polymers. Reproduced with permission.^[^ ^72^ ^]^ Copyright 2022, Nature. The conductive polymer acts as a scaffold for stem cells and provides electrical cues to guide their differentiation and integration into the host tissue. This approach aims to improve functional recovery after stroke by increasing the survival, differentiation, and integration of transplanted stem cells. This study revealed that electrical stimulation promoted significant motor function recovery and enhanced synaptic plasticity, highlighting the potential of this approach for treating stroke. B–I) Gene expression and neurite formation of encapsulated PC12 cells in GelMA/GO hydrogels. Reproduced with permission^[^ ^73^ ^]^ Copyright 2022, ACS. B,C) Gene expression analysis showing the fold change in GAP43 and βIII‐tubulin expression in PC12 cells encapsulated in GelMA and GelMA/GO hydrogels, with and without electrical stimulation. These data indicate that the combination of GO and electrical stimulation significantly increases the expression of these genes, which are key markers of neuronal differentiation. D‐G): Immunofluorescence images of encapsulated PC12 cells after 7 days of differentiation showing neurite formation (red: GAP43; green: βIII‐tubulin; blue: DAPI). Along with the neurite presence and length shown in (H) and (I), the images demonstrate that the presence of GO and electrical stimulation enhances neurite outgrowth and branching, indicating increased neuronal differentiation and network formation. Scale bars: 100 µm. J) Stable neural interface constructed by ICAA‐C. Reproduced with permission.^[^ ^74^ ^]^ Copyright 2024, Nature. Representative immunofluorescence images of vagus nerves explanted at 4 weeks postimplantation in the sham (top row), ICAA‐C (middle row), and commercial cuff (bottom row) surgery groups. The images are magnified ×45 (left) and ×175 (right) to show regions of the nerves. Scale bar, 20 µm (left), 10 µm (right). The ICAA‐C group showed successful nerve regeneration in the presence of neurofilaments (NFM, red) and Schwann cells (S100, green) and minimal macrophage infiltration (Iba‐1, green). In contrast, the commercial cuff group presented a reduced number of Schwann cells and increased macrophage infiltration, indicating nerve damage and inflammation.

In nerve regeneration, conductive polymers have shown significant promise because they provide a supportive and stimulatory environment for nerve cells. Oh et al.^[^ ^72^ ^]^ demonstrated that electrical modulation of transplanted stem cells via conductive polymers improved functional recovery in a rodent model of stroke. Researchers have reported that electrical stimulation enhances the differentiation and integration of transplanted stem cells, leading to improved motor function recovery. This study highlights the potential of combining electrical stimulation with stem cell transplantation to promote nerve regeneration and functional recovery after stroke. Mendes et al.^[^ ^73^ ^]^ integrated graphene oxide hydrogels with electrical stimulation to control the release of neurotrophic factors, which are key molecules that promote neuronal survival and growth. This combination enhanced axonal growth, highlighting the potential of combining electrical stimulation with biomaterial design. Moreover, to address the need for minimally invasive treatments, Yang et al.^[^ ^74^ ^]^ developed an injectable conductive hydrogel for long‐term electrical stimulation of neural tissues, offering a promising strategy for chronic neuromodulation and the treatment of neurological disorders. **Figure** **7** highlights the different ways in which conductive polymers and electrical stimulation are used to advance nerve regeneration therapies.

Oh et al.^[^ ^72^ ^]^ demonstrated in Figure 7A that electrical stimulation of transplanted stem cells via conductive polymers increased the expression of stanniocalcin‐2 (STC2), a protein that promotes neuroprotection and angiogenesis, leading to improved functional recovery in a rodent model of stroke. Researchers reported that electrical stimulation increased STC2 expression in transplanted cells, and this increase was correlated with enhanced motor function recovery and synaptic plasticity. These findings suggest that STC2 plays a crucial role in mediating the beneficial effects of electrical stimulation on stem cell integration and functional recovery after stroke, highlighting the potential of combining electrical stimulation with stem cell transplantation to promote nerve regeneration. Following, Mendes et al.,^[^ ^73^ ^]^ investigated in Figure 7B‐I the use of graphene oxide (GO) in gelatic methacryloyl (GelMA) hydrogels to control the release of nerve growth factor (NGF) and promote neuronal differentiation. Researchers have reported that the presence of GO in hydrogels significantly reduces NGF release, whereas electrical stimulation enhances NGF uptake. This combination of GO and electrical stimulation led to increased expression of neuronal markers (GAP43 and βIII‐tubulin) and enhanced neurite outgrowth in encapsulated PC12 cells, indicating improved neuronal differentiation and network formation. These findings suggest that integrating GO and electrical stimulation in GelMA hydrogels can create a more effective platform for nerve tissue engineering by controlling the release of neurotrophic factors and promoting neuronal differentiation. Finally, Yang et al.^[^ ^74^ ^]^ developed an injectable conductive hydrogel (ICAA) for chronic neuromodulation, aiming to address the challenges of interfacing with small and delicate nerves. Researchers have focused on enhancing the injectability, conductivity, and mechanical stability of hydrogels for long‐term use. They achieved this by incorporating a multifunctional molecular regulator, tannic acid (TA), which modulates gelation kinetics and enhances interactions within the conductive network. The ICAA hydrogel demonstrated minimal swelling, strong adhesion to tissues and device substrates, and high conductivity, enabling effective electrical coupling with fine nerves. In vivo experiments revealed that the ICAA‐C neural interface, which was constructed by injecting an ICAA hydrogel into commercial cuff electrodes, enabled chronic vagus nerve stimulation in a rat model of myocardial infarction. The ICAA‐C interface exhibited minimal tissue damage and inflammation, as evidenced by the presence of neurofilaments and Schwann cells and minimal macrophage infiltration, as shown in the immunofluorescence images. This stable and biocompatible interface allows effective vagus nerve stimulation, leading to reduced inflammation, inhibited sympathetic nerve activity, and decreased myocardial fibrosis, ultimately maintaining heart function. This study highlights the potential of injectable conductive hydrogels for chronic neuromodulation and their promising applications in treating various neurological disorders.

While CPs have shown significant promise in nerve regeneration, carbon nanotube‐hydrogel composites have also gained attention for their unique properties, such as their ability to support both neuronal differentiation and stable network activity. Liang et al.^[^ ^45^ ^]^ demonstrated that these composites not only promote the differentiation of neurons but also maintain stable network activity, which is crucial for the formation of functional neural networks. This ability to preserve network integrity while promoting differentiation makes CNT‒hydrogel composites particularly valuable for nerve regeneration.^[^ ^40^ ^]^ Similarly, Liu et al.^[^ ^75^ ^]^ used three‐dimensional electroconductive CNT‐based hydrogel scaffolds to enhance the differentiation of stem cells from the apical papilla, which is a promising source for neural regeneration. These scaffolds significantly improved stem cell differentiation into neurons, with the conductive properties of the carbon nanotubes supporting the formation of neural tissue. Graphene and its derivatives are also being explored in tissue engineering for their potential to create advanced neural scaffolds. Fabbri et al.^[^ ^76^ ^]^ demonstrated the ability of GO electrodes to electrically stimulate distinct calcium signals in brain astrocytes. This ability to influence cellular activity through electrical signals within a tissue‐engineered construct is critical for promoting functional integration and network formation. The incorporation of the conductivity of graphene into neural tissue scaffolds eases the creation of microenvironments that not only support cellular proliferation and differentiation but also promote the development of functional neural circuitry, thereby enhancing biological integration and long‐term efficacy.

Cardiac tissue engineering also benefits from electrical stimulation, as the electrical conductivity of engineered tissue is crucial for its function. Liang et al.^[^ ^45^ ^]^ investigated conductive PPy‐encapsulated silk fibroin fibers for cardiac tissue engineering. These fibers supported the formation of functional cardiac tissue, demonstrating significant improvements in cell alignment and electrical conductivity compared with nonconductive scaffolds. In fact, researchers have reported a 30% increase in cell alignment along conductive fibers, as evidenced by quantitative image analysis of aligned cardiomyocytes. This enhanced alignment is attributed to the contact guidance provided by the conductive PPy layer, which mimics the anisotropic nature of native cardiac tissue, directing cardiomyocyte elongation and organization. Furthermore, the conductive scaffolds presented a threefold increase in electrical conductivity, directly impacting the propagation of electrical signals within the engineered tissue. This enhanced conductivity is essential for promoting the growth and repair of heart tissue, as it facilitates synchronized contractions and functional integration, providing a promising approach for cardiac regeneration. These examples demonstrate the diverse applications of electrical stimulation in tissue engineering, showing the translational potential of electroactive materials in regenerative medicine, particularly for nerve and cardiac tissue repair.

#### 2.3.2 Drug Delivery

Recent advancements in electrically responsive drug delivery systems have led to the development of innovative materials designed to release therapeutic agents upon electrical stimulation. PPy‐coated polyvinylidene fluoride (PPy‐PVDF) fibers have been developed for the delivery of bioactive molecules such as basic fibroblast growth factor and nerve growth factor. These aligned electrospun fibers enable tunable release profiles through electrical stimulation, making them particularly suitable for musculoskeletal tissue regeneration.^[^ ^77^ ^]^ This ability arises from the ability to precisely control the release of growth factors, which is crucial in musculoskeletal regeneration, where different stages of healing require varying concentrations of these factors. For example, higher concentrations may be needed initially to stimulate cell proliferation and migration, whereas lower concentrations are preferred later to promote tissue maturation and remodeling. This temporal control over drug release, afforded by electrical stimulation, enhances the effectiveness of the therapy and minimizes potential side effects. Another promising platform is the electroresponsive conductive hydrogel patch, which incorporates silver nanowires (AgNWs) into a blend of alginate and GelMA. This combination enhances both electrical conductivity and mechanical properties, enabling controlled transdermal drug release.^[^ ^78^ ^]^ Conducting polymer hydrogels have also been explored for the electrically modulated release of proteins, providing a highly localized and tunable delivery method for therapeutic proteins. This localized delivery is particularly advantageous for treating conditions such as cancer or inflammation, where targeted delivery minimizes damage to surrounding healthy tissues. This strategy minimizes systemic side effects while maintaining the bioactivity of the released molecules.^[^ ^79^ ^]^

Together, these studies present the versatility of electrically responsive materials in drug delivery applications. The choice of conductive components, such as GO or AgNWs, significantly influences system performance, enabling precise control over drug release kinetics. These advancements have expanded implications for personalized medicine, allowing for the fine‐tuning of drug release in response to physiological needs, which can enhance treatment efficacy across various medical applications.

#### 2.3.3 Biosensing

Electrically responsive materials offer a unique platform for creating biosensors that can detect various biomolecules or physiological signals with high sensitivity and in real time. In the study by So et al.,^[^ ^80^ ^]^ electrical stimulation was used in combination with nanopatterns to influence cellular behavior, such as adhesion, proliferation, and differentiation. The electrical fields mimic biomimetic environments, enabling real‐time detection of changes in cellular responses. The nanopatterned surfaces were fabricated via UV‐curable polyurethane acrylate (PUA) on a polyethylene terephthalate (PET) film via UV nanoimprint lithography. The biosensing platform uses these electrical properties to monitor cellular activities in response to electrical signals, resulting in high sensitivity and potential for continuous monitoring in tissue engineering applications. This study shows how electrical stimulation can be employed for biosensing at the cellular level.

Similarly, Ouyang et al.^[^ ^81^ ^]^ used electrical stimulation for neural biosensing. The core material used in the study is a magnetoelectric (ME) composite including a piezoelectric layer made of lead zirconate titanate (PZT) and a magnetostrictive layer made of Metglas. The implant records neural signals and provides real‐time stimulation on the basis of the data obtained from biosensors embedded within the device. This platform detects changes in neural activity and triggers electrical stimulation or drug delivery, enabling real‐time feedback and continuous monitoring of brain activity. This closed‐loop system demonstrates how electrical stimulation and biosensing can be integrated for neural modulation. Both studies highlight the versatility of electrically responsive materials as effective platforms for biosensing, measuring changes in electrical properties, whether through cellular behavior or neural signals, and enabling real‐time feedback via electrical stimulation. These systems offer high sensitivity, continuous tracking, and potential applications in both cellular and neural biosensing.

#### 2.3.4 Other Applications

In addition to the key areas discussed above, electrically responsive materials hold promise in various other biomedical applications. For example, electrical stimulation accelerates wound healing, and electrically responsive materials can be incorporated into wound dressings to promote tissue regeneration.^[^ ^82^ ^]^ One example is the use of conductive hydrogels, which can deliver electrical stimulation to the wound site and promote cell proliferation and migration. In neural interfaces, electrically conductive materials are crucial for creating implants that can record or stimulate neural activity, leading to potential treatments for neurological disorders and the development of advanced prosthetics. For example, conductive polymers can be used to coat electrodes for neural stimulation, improving their performance and stability. Furthermore, electrically responsive materials can be used to create bioactuators, such as artificial muscles or other devices that generate mechanical motion, offering new possibilities for assistive technologies and implantable devices. Carbon nanotubes, for instance, can be incorporated into hydrogels to create bioactuators that respond to electrical signals and generate mechanical motion.^[^ ^83^ ^]^

### 2.4 Recent Advances

The field of electrically responsive materials for biomedical applications is rapidly evolving, with researchers constantly exploring innovative designs and applications. Recent advances in material design include self‐healing hydrogels that can repair themselves after damage, improving their durability for drug delivery and tissue engineering.^[^ ^84^ ^]^ New Cs with improved biocompatibility and biodegradability have been synthesized, reducing the risk of adverse reactions and enabling their use in implantable devices. Advances in materials science and fabrication techniques have led to the development of flexible and stretchable electronics that can conform to the complex shapes of the body, opening new possibilities for wearable sensors and implantable devices. Furthermore, 3D printing techniques are being used to create complex and customized structures from electrically responsive materials, enabling the fabrication of personalized medical devices and implants.^[^ ^72^ ^]^

These advancements in materials have opened a wide range of possibilities, such as the development of brain‒computer interfaces that can record and stimulate neural activity, offering potential for treating neurological disorders and restoring lost function. These materials are also being incorporated into smart drug delivery systems that can release drugs in a controlled and targeted manner, improving the efficacy and safety of drug therapies. Flexible and stretchable electronics are being used to create wearable biosensors that can continuously monitor physiological signals, such as heart rate, body temperature, and sweat composition, providing valuable health information. Furthermore, electrically responsive materials are being used to develop implantable devices, such as pacemakers, neurostimulators, and drug delivery implants, that can be controlled wirelessly and provide personalized therapies.^[^ ^85^ ^]^

In addition to the development of new materials and applications, researchers have focused on improving the biocompatibility and performance of existing electrically responsive materials. Surface modification techniques, such as coating materials with biocompatible polymers or functionalizing them with bioactive molecules, are being used to increase their interaction with biological systems and reduce the risk of adverse reactions. Nanostructured materials, such as nanowires and nanoparticles, are being incorporated to enhance the electrical and mechanical properties of these materials, leading to improved performance in biomedical applications. Furthermore, computational modeling is being used to design and optimize electrically responsive materials for specific biomedical applications, leading to improved performance and reduced development time.^[^ ^86^ ^]^

### 2.5 Challenges and Future Directions

While electrically responsive materials hold great promise for biomedical applications, several challenges need to be addressed to fully realize their potential. Ensuring long‐term biocompatibility and safety is crucial, as some materials may be toxic or induce an immune response, leading to inflammation, fibrosis, or rejection of the implant. In addition, eliminating surface adsorption of biological compounds is a key challenge to avoid a reduction in the electrical performance of these materials due to fouling. Strategies such as surface modification with biocompatible polymers, such as polyethylene glycol (PEG) or hyaluronic acid, and the use of naturally derived materials are being explored to mitigate these risks. While these approaches improve the biocompatibility and stability of electrically responsive materials, they also reduce the electrical performance of the material. This novel work, which utilizes the unique properties of the glycoprotein lubricin, shows significant promise in overcoming these listed challenges while maintaining the desired electrical performance.^[^ ^87^, ^88^ ^]^

Improving the stability of these materials is essential, as they can degrade or lose their properties over time due to factors such as oxidation, hydrolysis, or mechanical stress in complex biological environments. Achieving sufficient conductivity for applications such as neural interfaces and biosensors remains a challenge, demanding research into new materials with higher conductivity, such as graphene and CNTs, and fabrication techniques that optimize their electrical properties.^[^ ^89^ ^]^ Manufacturing complex electrically responsive devices with precise control over their structure and properties is also difficult and requires advances in microfabrication and nanofabrication techniques, such as 3D printing and electrospinning, to create more intricate and functional devices. Another challenge is the integration of these materials with biological tissues, as the interface between the material and the tissue can significantly affect the performance of the device and its longevity. Strategies such as surface modification with cell‐adhesive peptides or growth factors are being investigated to improve tissue integration. Finally, translating promising in vitro results to in vivo applications is often challenging due to the complex biological environment and factors such as biofouling, where proteins and other biomolecules adsorb to the material surface, immune responses, and material degradation. Addressing these challenges will require a multidisciplinary approach, combining expertise in materials science, engineering, biology, and medicine, to develop safe, effective, and reliable electrically responsive materials for a wide range of biomedical applications.^[^ ^90^ ^]^

## 3 Optically Responsive

While electrical stimuli excel in temporal precision and bioelectrical compatibility, they are often limited by the need for direct contact or implanted electrodes. This limitation drives us to consider optical stimuli, which offer unparalleled spatial resolution and the ability to deliver energy remotely through tissue. Light‐responsive materials provide a noninvasive alternative that can achieve highly localized effects, although with different penetration characteristics and activation mechanisms than their electrical counterparts.

In this section, we focus on those materials that can respond to light in a way that generates a useful outcome, such as the control or manipulation of biological and pharmacological processes. However, materials that can generate a secondary optical observable will also be briefly discussed in the context of imaging and contrast agents. Manipulation and control generally rely on the conversion of optical energy into different forms, including photothermal, photovoltaic, photomechanical, and photochemical transduction processes,^[^ ^91^, ^92^, ^93^, ^94^ ^]^ whereas imaging agents rely on phenomena such as elastic and inelastic light scattering, absorption, birefringence, and nonlinear optical processes.^[^ ^95^, ^96^ ^]^ We note that light is known to have a direct effect on biology via a number of intrinsic mechanisms,^[^ ^97^ ^]^ but these photobiological effects will not be considered further here, where the focus is on engineered biomaterials as extrinsic photoresponsive interfaces. Similarly, the direct photomechanical interactions that can be achieved with intense ultrafast laser pulses are not considered here.^[^ ^98^ ^]^

While the optical transduction processes listed above are all reasonably well understood, multiple processes can, in principle, occur simultaneously, which can make it difficult to identify the primary mechanism underlying the resulting action. In particular, all transduction processes require absorption of the incident light before charge transfer, stress wave propagation, or chemical reactions can occur. Any excess photon energy would therefore be expected to result in an additional thermal response. Therefore, it is important to keep the potential for mechanistic ambiguity in mind when interpreting results.

A wide range of photoresponsive biomaterials has been developed for diverse applications, such as tissue regeneration, controlled release, the modulation of cellular responses, and photodynamic therapies. The important categories of photoresponsive biomaterials include the following:

- Hydrogels that undergo morphological alterations or can be optically crosslinked or degraded.^[^ ^99^ ^]^
- Liquid crystal biomaterials that offer a wide range of both optical sensitivities and optical responses to various stimuli.^[^ ^100^ ^]^
- Transparent conducting oxides, primarily indium tin oxide (ITO).^[^ ^101^ ^]^
- Nanoparticles (NPs),^[^ ^102^ ^]^ including plasmonic nanoparticles (typically gold), quantum dots,^[^ ^103^ ^]^ polymer beads,^[^ ^104^ ^]^ upconversion nanoparticles,^[^ ^105^ ^]^ photovoltaic nanoparticles (e.g., CuS),^[^ ^106^ ^]^ and carbon dots.^[^ ^107^ ^]^
- 2D nanomaterials such as transition metal dichalcogenides, transition metal oxides, and conducting polymer nanosheets.^[^ ^108^ ^]^

Combinations of the materials listed above have also been widely studied, as composite photoresponsive materials can provide multiple functionalities. For example, the thermoresponsive acrylic acid poly(N‐isopropylacrylamide) (PNIPAM) hydrogel has a critical solution temperature close to physiological temperature and has been combined with photothermal gold nanoparticles (Au NPs) for drug release applications.^[^ ^109^ ^]^ In another example, soft GelMA containing electrically responsive graphene oxide conjugated with photoresponsive gold nanorods (NRs) has been used to match the mechanical stiffness properties and deliver photothermal stimulation to explanted rat retinal tissue.^[^ ^110^ ^]^

**Figure 8:**
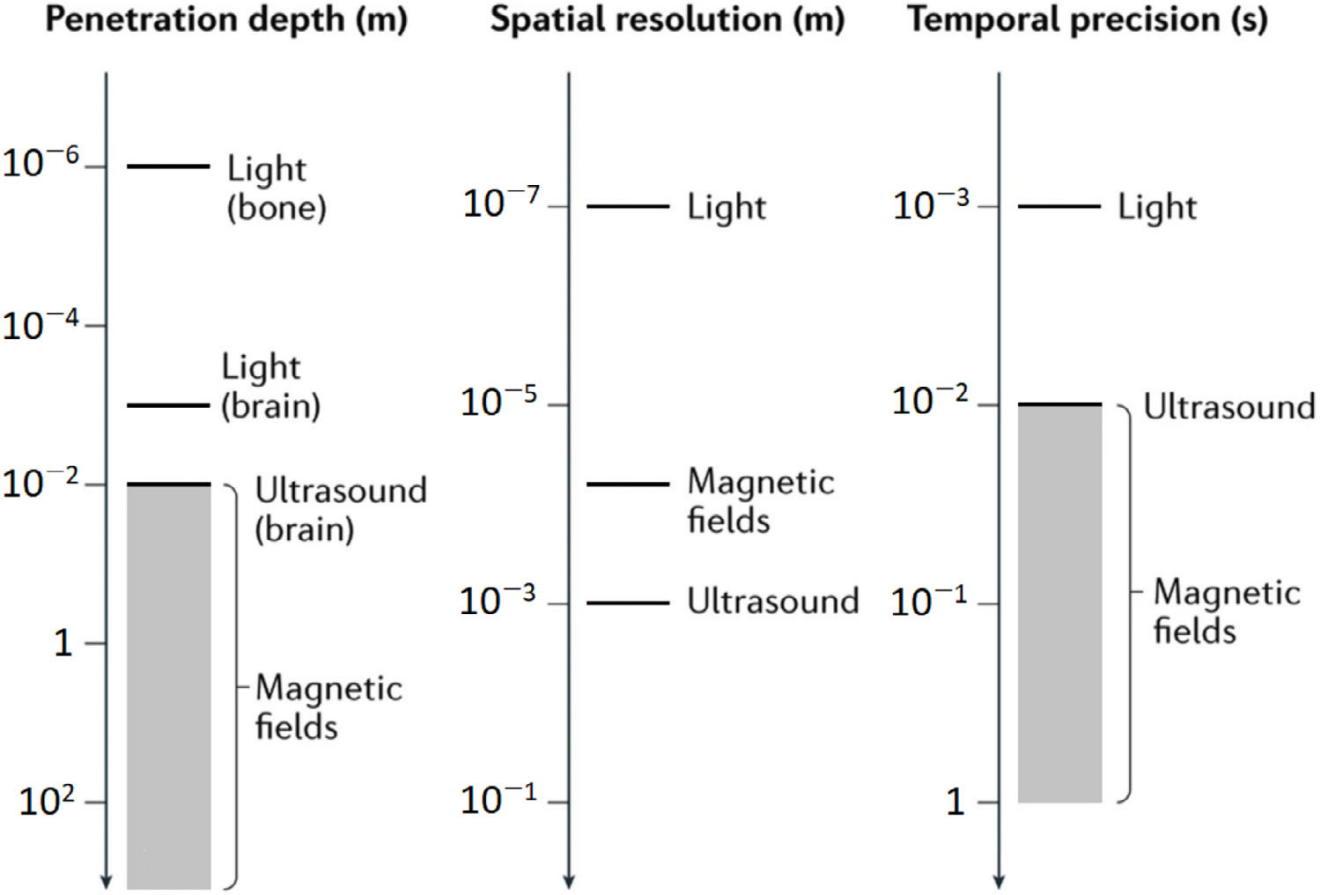
Comparison of tissue penetration depth, spatial resolution, and temporal precision for ultrasonic, optical, and magnetic stimuli.

As summarized in **Figure** **8** below, light‐based stimuli offer greater spatial resolution and temporal precision than do ultrasound or magnetic fields but have a smaller penetration depth in tissue. The actual depth is highly dependent on the wavelength and type of tissue: blue and UV wavelengths may only penetrate on the order of micrometres owing to absorption and scattering, whereas wavelengths in the first (650–900 nm) and second (1000–1700 nm) near infrared (NIR) windows may penetrate on the order of millimeters. Hemoglobin is a major contributor to absorption at visible wavelengths, whereas the strong overtone absorption of water at 1400–1500 nm tends to interrupt the transmission of light in the NIR‐II window. Shorter wavelengths are also more likely to generate autofluorescent backgrounds in tissue, whereas both scattering and background interference are generally reduced at longer wavelengths.^[^ ^111^ ^]^ The advantages of the second NIR window have generated significant interest in the development and application of materials that respond to optical stimuli in this wavelength range.^[^ ^112^ ^]^ Despite the overall decreasing trend at longer wavelengths, the scattering coefficient is also tissue dependent, with skin scattering relatively strong in the visible region compared with other tissues. At longer NIR wavelengths, skin, bone, and fatty tissue tend to scatter more than the brain and other soft tissues do.^[^ ^113^ ^]^ On the other hand, some specialized tissues, particularly those in the mammalian eye, can be highly transmissive, making the retina an outstanding target for photoresponsive biomaterials.^[^ ^114^ ^]^

Given the wide range of materials and response mechanisms described above, the following examples of applications are necessarily incomplete and only aim to provide a broad sample of the field.

### 3.1 Applications

#### 3.1.1 Tissue Engineering

Bioengineered tissues utilize extracellular matrix (ECM) mimics to cultivate cells in a 3D environment. The tunability of hydrogel properties, such as stiffness and pore size, not only facilitates the growth of specific cell types and behaviors but can also contribute to the development of optically responsive materials. For example, the accumulation of Au NPs within a hydrogel's pores has been used to establish a surface‐enhanced Raman scattering (SERS)‐active hydrogel for the measurement of cellular markers that diffuse near these pores.^[^ ^115^ ^]^ Additionally, the application of Au NPs within hydrogel pores was illustrated by Eom et al. in the creation of alveolar (lung) mimic tissue for the growth and measurement of lung cells.^[^ ^116^ ^]^ They also incorporated cytochrome c to allow the detection of oxygen. SERS signals from cultured cells were recorded via 785 nm excitation, revealing a novel method to monitor cellular release through Raman fingerprints.

Gold nanoparticles and other optically responsive biomaterials can also be used to promote peripheral nerve regeneration.^[^ ^3^ ^]^ NIR exposure of gold nanorods (Au NRs) has been shown to stimulate neurite outgrowth in NG108‐15 neuronal cells.^[^ ^117^ ^]^ The greatest outgrowth was observed after the endocytosed particles were illuminated with the highest laser dose (7.5 W cm^−2^), resulting in an average increase in neurite length of almost 36% compared with that of the nonirradiated sample. More recently, Qu et al. reported that circularly polarized photons can accelerate the differentiation of neural stem cells into neurons when DNA‐bridged chiral assemblies of gold nanoparticles are entangled with the cells’ cytoskeletal fibers. It appears that the nanoparticle assemblies exert a circularly polarized light‐dependent force on the cytoskeleton, and the resulting light‐induced periodic mechanical deformation of the actin nanofibers with a frequency of 50 Hz promotes differentiation. Interestingly, when neural stem cells were implanted in the hippocampus of a mouse model of Alzheimer's disease and illuminated with a polarity‐optimized protocol, the formation of amyloid plaques was reduced by more than 70%.^[^ ^118^ ^]^

Nanodiamonds with nitrogen‐vacancy (NV) color centers embedded in hydrogels have been investigated for their use in quantum sensing with three‐dimensional cultured cells. These optically active nanodiamonds are integrated into thermoresponsive poly(N‐isopropylacrylamide) (pNIPAM) hydrogels that also contain magnetic NPs.^[^ ^119^ ^]^ This arrangement creates variations in distance in response to changes in the hydrogel volume associated with temperature shifts. Furthermore, a silk hydrogel infused with nanodiamonds was employed to study neural cells in 3D,^[^ ^120^ ^]^ with nanodiamond alignment acting as a marker for neural growth.

#### 3.1.2 Drug Delivery

**Figure 9:**
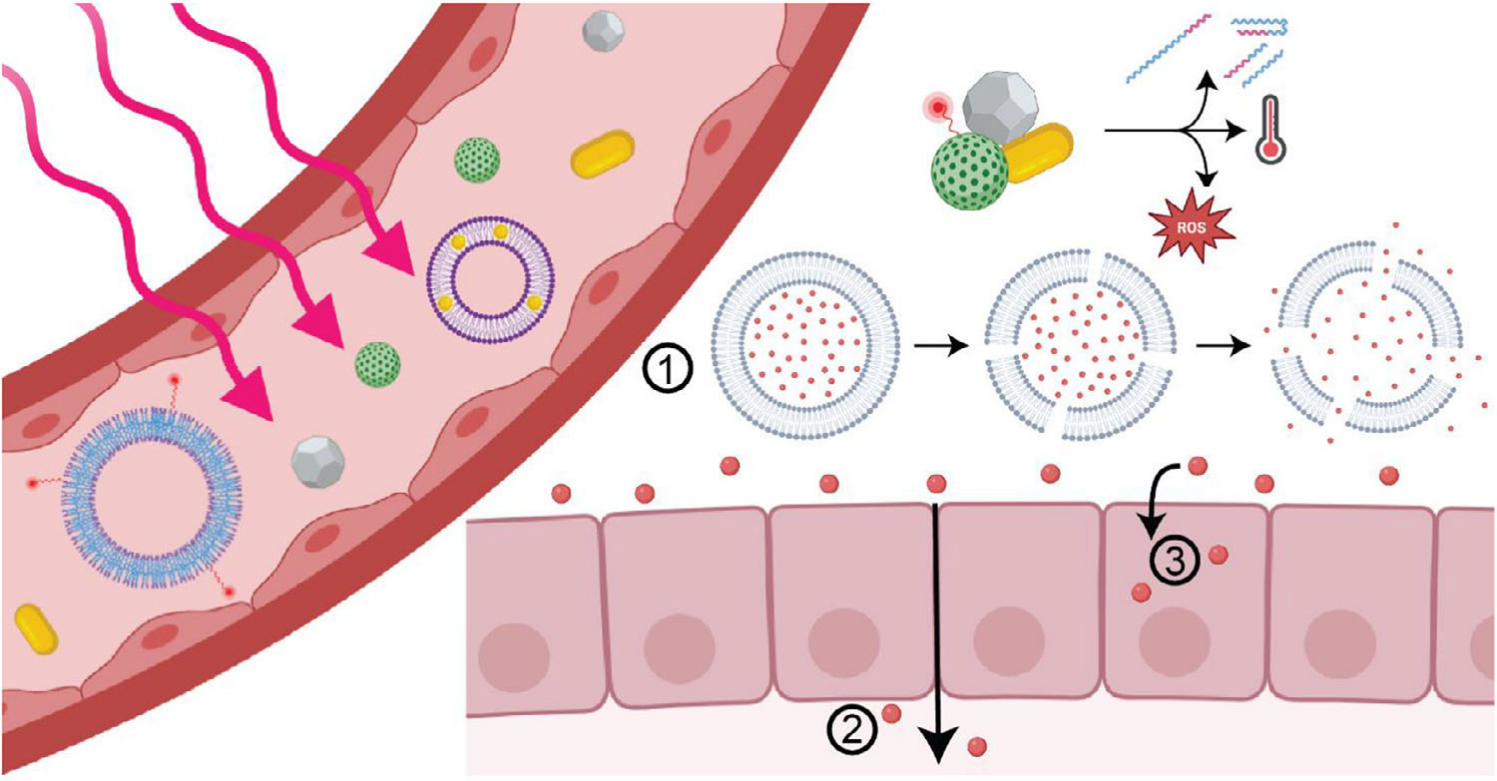
Conceptual illustration of optically responsive nanomaterials in drug delivery applications. Upon optical stimulation, these materials can generate heat, produce reactive oxygen species (ROS), or induce conformational or structural changes in surrounding lipids or polymeric drug carriers. 1) Payload delivery: Optical activation triggers the breakdown of drug‐loaded liposomes or polymersomes, enabling targeted and localized drug release. 2) Paracellular transport: Photothermal stimulation enhances drug transport by loosening tight junctions or promoting transcellular passage across biological barriers. 3) Optoporation: Localized heat or photomechanical effects transiently increase cell membrane permeability, facilitating the intracellular delivery of drugs, genes, or viral vectors. Created via Biorender.com.

A major application of optically responsive nanomaterials in drug delivery is controlled payload release. Among the most widely studied systems are liposomes and polymersomes, where lipids or polymers form nanostructured vesicles capable of encapsulating hydrophilic or hydrophobic drugs.^[^ ^34^ ^]^ Under light exposure, optically responsive nanomaterials within or attached to vesicles trigger membrane destabilization, causing controlled drug release.^[^ ^121^ ^]^ The associated mechanisms for the modulation of membrane permeability and barrier transport are conceptually illustrated in **Figure** **9**.

Various optically responsive nanomaterials have been explored for this purpose. A photothermal approach, as demonstrated by Yi et al.,^[^ ^122^ ^]^ incorporates indocyanine green (ICG) molecules into the liposome membrane. Upon NIR irradiation, the local temperature increased, leading to membrane destabilization and subsequent drug release. Additionally, the photodynamic properties of ICG facilitate the conversion of an encapsulated prodrug into its active form. An alternative photothermal approach relies on functionalizing liposomes with thermosensitive gold nanoprisms so that plasmonic heating upon localized NIR exposure triggers the breakdown of the liposomal membrane, enabling targeted drug delivery.^[^ ^123^ ^]^ Another study used amphiphilic porphyrins embedded in drug‐loaded liposomes, where the generation of reactive oxygen species (ROS) upon NIR exposure led to lipid oxidation, ultimately degrading the carrier and releasing the drug.^[^ ^124^ ^]^ Similarly, Zheng et al.^[^ ^125^ ^]^ developed an allosteric polymersome system in which photosensitizer molecules (IR780) embedded in the polymer membrane generated ROS upon NIR irradiation, inducing a conformational shift in the polymer structure and increasing membrane permeability.

Optically responsive nanomaterials can also be used to promote the delivery of drugs across otherwise impermeable barriers. This usually involves increasing or enabling the paracellular/transcellular transport of chemicals to allow broad delivery to isolated organ systems such as the brain.^[^ ^99^, ^126^ ^]^ In this area, pulsed laser excitation of vascular‐targeted spherical Au NPs has been used to increase the penetration of paclitaxel across multiple blood‒brain barrier models in mice.^[^ ^127^ ^]^ Similarly, Jin et al.^[^ ^128^ ^]^ increased the payload delivery of antidepressant drugs across the blood–brain barrier through the photothermal effect generated by black phosphorus nanosheets exposed to NIR light.

Additionally, NPs can enhance cell membrane permeability, enabling the precise delivery of payloads via nonviral transfection methods. This effect is often mediated by transient photothermal interactions, which temporarily disrupt the cell membrane and facilitate intracellular delivery. Pylaev et al.^[^ ^129^ ^]^ developed an optoporation system in cell cultures mediated by the near‐infrared irradiation of a gold nanostar layer. Their study demonstrated successful payload delivery of various sizes and enabled targeted optoporation in individually selected cells. In other work, porous silicon NPs were used for the localized transfection of mRNA in organoids via two‐photon NIR stimulation.^[^ ^130^ ^]^ Expanding the scope to in vivo applications, Wilson et al.^[^ ^131^ ^]^ conducted a proof‐of‐concept study demonstrating that femtosecond NIR laser irradiation of functionalized Au NRs enabled the effective delivery of macromolecules into retinal ganglion cells.

#### 3.1.3 Imaging

While the use of optically responsive materials in drug delivery represents a rapidly evolving and innovative application, their role in imaging is relatively well established. Most commonly, these materials have been employed as fluorescent probes in optical imaging because of their bright and stable emission properties. However, recent advances have expanded their use into other modalities, with increasing interest in their application as contrast agents for photoacoustic, X‐ray, and ultrasound imaging. These techniques rely on alternative physical mechanisms such as optical‐to‐acoustic conversion or X‐ray attenuation to enable imaging at greater tissue depths and in clinically relevant settings. The following section highlights both established and emerging imaging strategies that diversify the role of optically responsive materials in biomedical imaging.

Fluorescent nanodiamonds represent a class of optically responsive nanomaterials that can be used as fluorescent probes for imaging applications with relatively high brightness and optical stability. Variations in defects within the nanodiamond can lead to a diverse array of fluorescent particles, which are typically imaged via confocal microscopy.^[^ ^132^ ^]^ However, these particles may also be assessed through two‐photon absorption techniques, including multiphoton microscopy, which offers advantages for detecting light emission from the particles within thicker tissue samples. Nanodiamonds have traditionally been employed for cell tracking and internalization studies.^[^ ^133^ ^]^ For example, a study by Morita et al.^[^ ^134^ ^]^ used antibody‐labeled fluorescent nanodiamonds to mark the nucleus of yeast cells. A notable advantage of nanodiamonds in cellular applications is their comparatively low toxicity. For example, studies on cell tracking have demonstrated that nanodiamonds are easily internalized and effectively monitor cellular behavior in culture environments.^[^ ^135^, ^136^ ^]^ Furthermore, the size of nanodiamonds does not significantly affect cellular responses,^[^ ^137^, ^138^ ^]^ and carboxylated nanodiamonds have negligible toxic effects on various cell lines.^[^ ^139^, ^140^ ^]^ A challenge with nanodiamonds is that their color intensity and fluorescence brightness hinge on the number of defects.^[^ ^95^ ^]^ This results in variations in the defect populations and, consequently, differences in brightness among the particles.

Darkfield microscopy is commonly used to image Au NPs and detect particle internalization within cells.^[^ ^141^, ^142^ ^]^ However, challenges arise when attempting to detect these particles in thicker tissue samples, primarily due to limitations in optical sectioning due to scattering in the tissue itself. Multiphoton microscopy is preferred for visualizing large tissue samples down to subcellular regions. This technique provides optical sectioning, enabling deeper penetration into samples than conventional confocal methods while also reducing photobleaching effects. Compared with nanotriangles, cubes, and rods, gold nanoparticles exhibit two‐photon absorption characteristics, with nanospheres demonstrating the lowest two‐photon luminescence (TPL).^[^ ^143^ ^]^ In contrast, Au NRs show greater potential, with two‐photon cross sections reaching ≈42 000 GM, in contrast to the 83 GM for spheres.^[^ ^144^ ^]^ Furthermore, nanorods can be optimized for near‐infrared wavelengths, thus minimizing tissue absorption.

Gold nanoparticles can also be used as contrast agents in clinical imaging applications, including computed tomography (CT) and X‐ray imaging. This application is mainly due to their X‐ray attenuation, pharmacokinetics, and biodistribution properties.^[^ ^145^ ^]^ Compared with traditional iodine‐based contrast agents, the higher atomic number (density) and attenuation of gold make it a better option for CT and X‐ray imaging applications.^[^ ^146^, ^147^ ^]^ Research exploring the effectiveness of Au NPs as contrast agents in cancer studies using both in vivo and in vitro models has shown that labeled Au NPs can help identify the locations of cancerous tissues. Bulent et al. employed a glucose analog to label Au NPs, achieving targeted delivery to cancer cells within a human lung cancer model.^[^ ^148^ ^]^ Moreover, imaging cardiac disease via CT and Au NP contrast agents has been demonstrated through the application of particles functionalized with a known targeting ligand for cardiac fibrosis.^[^ ^149^ ^]^

Finally, the thermal response of NPs and molecular dyes, when labeled with antibodies or ligands, can be used in photoacoustic and photothermal imaging modalities to monitor specific structures or molecular processes. Photoacoustic imaging relies on the conversion of absorbed optical energy to ultrasound emission,^[^ ^150^ ^]^ whereas photothermal imaging generates a thermal lens that can be detected by a second probe beam.^[^ ^151^ ^]^ In combination with the previously described stimulus‐responsive modalities, these imaging modalities open up numerous theranostic applications that combine diagnostic imaging with therapeutic interventions.^[^ ^152^, ^153^ ^]^

#### 3.1.4 Therapeutic Interventions

Modulation of the biological environment by optically responsive NPs is one of the simplest and most direct applications in the regulation of local temperature. This photothermal effect arises from mechanisms such as localized surface plasmon resonance, nonradiative relaxation, and thermoelastic vibrations, depending on the material system. A wide array of nanomaterials exhibit these responses, and their photothermal properties are harnessed in a broad range of applications.^[^ ^154^, ^155^ ^]^ One of the most prominent implementations of this phenomenon is in photothermal therapy (PTT), where light‐induced heating leads to targeted cell stimulation or damage. Among these materials, plasmonic NPs, particularly those composed of gold and silver, are widely studied because of their tunable optical properties and high photothermal conversion efficiency. The shape, size, and surface functionalization of these NPs critically influence their absorption characteristics and therapeutic efficacy.^[^ ^156^, ^157^ ^]^ A recent comparative study by Liu et al.^[^ ^158^ ^]^ investigated the performance differences between Au NRs and nanobipyramids, with the latter showing an improved photothermal conversion rate and two‐photon emission in liver cancer cells. Arami et al. used Au nanostars for the photothermal treatment of brain tumors in freely behaving mice,^[^ ^159^ ^]^ whereas immunoglobin G‐functionalized Ag NPs have been used for in vitro photothermal treatment of pancreatic cancer.^[^ ^160^ ^]^

Other examples of nonplasmonic NPs used for photothermal therapy include copper sulfide nanoparticles,^[^ ^161^ ^]^ MXenes,^[^ ^162^ ^]^ and black phosphorous nanosheets,^[^ ^163^ ^]^ among others. Recently, Xue et al.^[^ ^164^ ^]^ combined MXene nanosheets, bioinspired hydroxyapatite nanoflowers, and a gelatin methacryloyl/methacrylated hyaluronic acid (GelMA/HAMA) double‐network hydrogel to form an injectable near‐infrared responsive bone cement for diabetic fracture repair. The high photothermal conversion efficiency of the MXene was used to induce mild hyperthermia (40–43 °C), which, in combination with the sustained release of osteogenic ions from the hydroxyapatite nanoflowers, significantly increased the expression of markers for angiogenesis and osteogenic differentiation. Complete cortical bone remodeling was observed within 8 weeks.

**Figure 10:**
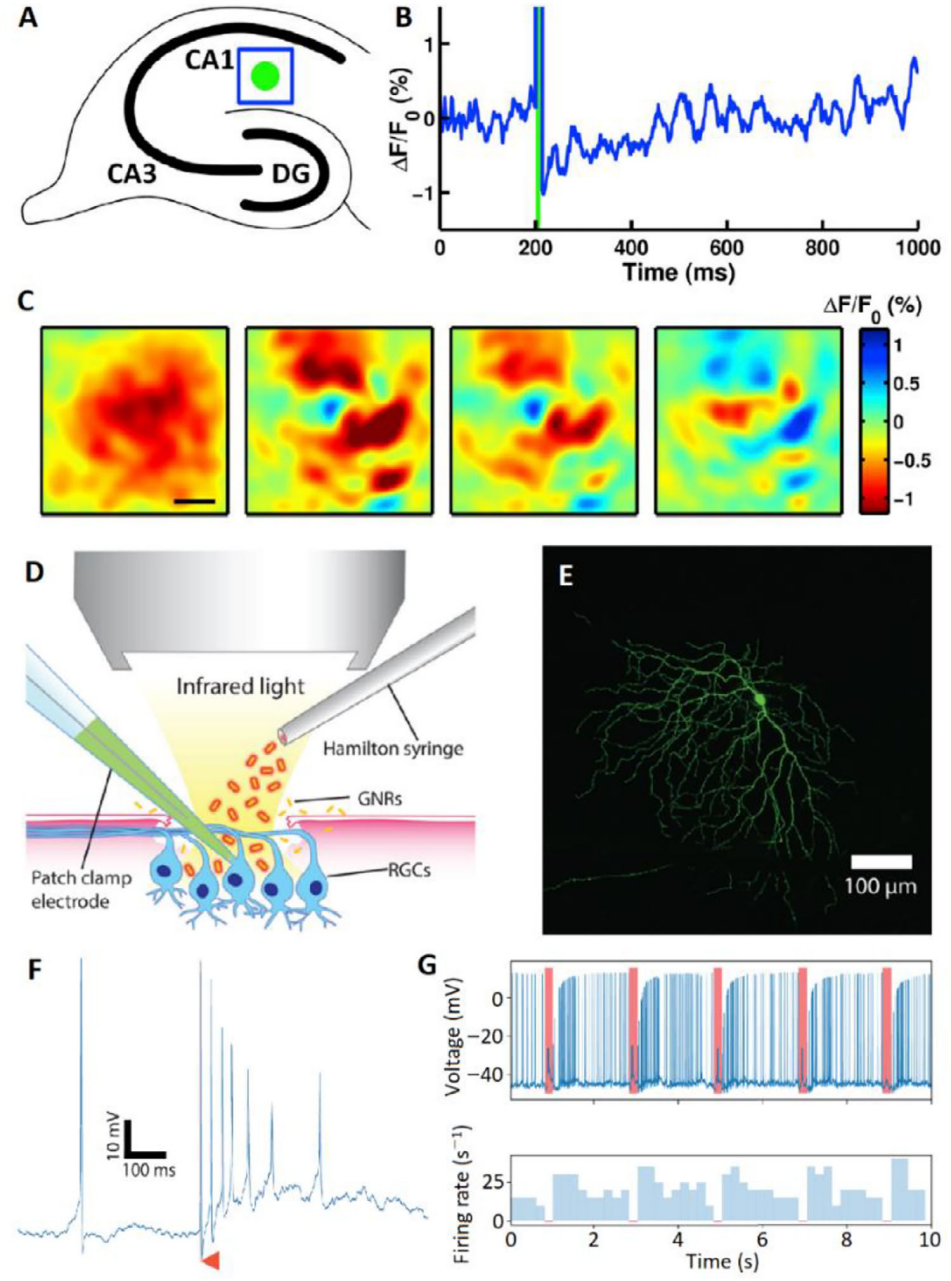
A) Approximate injection location (green dots) of AuNPs conjugated with the scorpion toxin Ts1 to target voltage‐gated sodium channels in the mouse hippocampus and the imaging region (blue square) in the CA1 region. B) Neuronal activity in response to a 532 nm optical stimulus (green bar) was monitored by means of fluorescence from the voltage‐sensitive infrared dye indocyanine green, revealing initial cellular depolarization followed by recovery to baseline over several hundred milliseconds. C) Spatially resolved patterns of neural activity induced by the optical stimulus were tracked over time as activity returned to baseline levels. This series of baseline‐subtracted images shows the average activity over four consecutive 80 ms windows starting 20 ms after the laser pulse. Scale bar = 100 µm. Reproduced with permission.^[^ ^1^ ^]^ Copyright 2015, Cell Press. D) Illustration of nanorod and NIR light delivery to retinal ganglion cells (RGCs) in explanted rat tissue. After a cell was patch clamped, the Au NRs were injected directly above a rip in the inner limiting membrane. E) Confocal fluorescence image of a single RGC after patching, clearly showing an intact soma, axon, and dendrites. F) An example of tonic spiking in response to a 500 *µs* laser pulse, with the red arrow indicating the timing of laser onset. G) Longer 200 ms laser pulses significantly and reversibly reduce the amplitude of action potentials over their entire duration (top; laser illumination periods marked by red bars). Time histogram of the corresponding spike rate, excluding spikelets with peak heights of less than −20 mV (bottom; bin width equal to pulse duration). Reproduced with permission.^[^ ^166^ ^]^ Copyright 2023. Wiley.

The photothermal properties of gold nanoparticles have also been proven to be well‐suited to the modulation of nerve electrical activity, as illustrated in **Figure** **10**. Yong et al.^[^ ^165^ ^]^ provided the first experimental demonstration of this effect, using endocytosed Au NRs to stimulate cultured rat primary auditory neurons with 780 nm illumination. The laser‐induced action potentials were associated with transient temperature increases of ≈6 °C. Subsequent work demonstrated that functionalized Au NPs could be used to target specific ion channels in the membrane of dorsal root ganglion neurons and mouse hippocampal brain slices. The particles formed stable associations with the cultured neurons without impeding their excitatory ability and generated optically evoked action potentials at relatively low NP concentrations.^[^ ^1^ ^]^ Importantly, this relatively noninvasive approach allows both stimulation and inhibition of nerve electrical activity with high spatial precision, which cannot be readily achieved through conventional electrical stimulation.^[^ ^166^ ^]^ The underlying thermal mechanisms are now reasonably well understood, and this approach is attracting interest as a potential basis for prosthetic vision,^[^ ^114^ ^]^ with one recent study delivering antibody‐conjugated AuNRs to bipolar cells in the mouse retina by intravitreal injection. A scanning NIR laser beam with a spot size of 20 µm was then shown to consistently evoke electrical activity in the visual cortex of both wild‐type and fully blind mouse models without inducing systemic toxicity or significant retinal damage.^[^ ^167^ ^]^

While PTT allows direct heat‐induced treatment via an optical stimulus, NPs can also be used to monitor the locally induced temperature via a theranostic hybrid approach. Here, temperature‐sensitive NPs can be covalently coupled to the previously mentioned photothermal nanomaterials. Several classes of nanomaterials, including upconversion nanoparticles (UCNPs),^[^ ^168^ ^]^ plasmonic nanocrystals,^[^ ^169^ ^]^ semiconductor quantum dots,^[^ ^170^, ^171^ ^]^ and graphene‐based materials,^[^ ^172^ ^]^ have been widely explored for high‐sensitivity optical temperature sensing. Zhu et al.^[^ ^173^ ^]^ described carbon‐coated lanthanide‐doped UCNPs for simultaneous photothermal therapy and temperature monitoring, resulting in increased spatial resolution of the therapy with less damage to surrounding normal tissue.

In addition to temperature modulation, optically activated nanomaterials play a pivotal role in driving light‐induced chemical reactions, particularly in photodynamic therapy (PDT).^[^ ^174^ ^]^ In this process, photoexcitation of the nanomaterial leads to the generation of reactive oxygen species (ROS), which can cause localized cytotoxicity that is useful for therapeutic applications, most notably in cancer treatment.^[^ ^175^ ^]^ The mechanism involves the absorption of light by a photosensitizer, which promotes the transition of an electron to an excited singlet state. Through intersystem crossing, the electron transitions to a triplet state, where it can transfer energy either to nearby biomolecules (Type I reactions) or to molecular oxygen (Type II), resulting in the production of ROS such as singlet oxygen or hydroxyl radicals.^[^ ^176^ ^]^

Nanomaterials that can act directly as photosensitizers include titanium dioxide (TiO_2_) and zinc oxide (ZnO) nanoparticles,^[^ ^177^ ^]^ which generate ROS under UV or visible light. More recently, efforts have been made to make these NPs more efficient by forming complex composite structures. Notably, Gilson et al.^[^ ^178^ ^]^ developed a ruthenium complex, a TiO_2_ NP conjugate capable of generating large amounts of ROS under hypoxic conditions. In other work, Jardon‐Guadarrama et al. combined TiO_2_ NPs functionalized with folic acid with zinc phthalocyanine, increasing the light absorption spectrum in the visible range.^[^ ^179^ ^]^

Carbon‐based materials such as carbon quantum dots, fullerenes, and graphene oxide have also been shown to have photosensitizing qualities.^[^ ^180^, ^181^, ^182^, ^183^ ^]^ Xu et al.^[^ ^184^ ^]^ used iodine‐ and nitrogen‐doped carbon dots for photodynamic therapy and tumor growth inhibition in in vivo studies in mice. In addition to serving as photosensitizers themselves, nanomaterials can also function as photosensitizer carriers, facilitating energy transfer through mechanisms such as Förster resonance energy transfer (FRET) or wavelength conversion. UCNPs are particularly advantageous in this context, as they can convert near‐infrared (NIR) light to higher‐energy UV or visible light, which is more effective at exciting conventional photosensitizers.^[^ ^185^ ^]^

Zuo et al.^[^ ^186^ ^]^ developed photoswitchable UCNPs coupled to the previously mentioned TiO_2_ nanoparticles. Near‐infrared irradiation at set wavelengths (800 nm/980 nm) inhibits or activates the UV emission of UCNPs. The corresponding activation of the coupled TiO_2_ NPs enables their use in deep tissue photodynamic therapy. Quantum dots and Au NPs can play similar roles via FRET mechanisms.^[^ ^187^, ^188^ ^]^

Optically responsive nanomaterials can also be employed to modulate the activity of excitable biological cells through the formation of dipoles.^[^ ^189^, ^190^ ^]^ This technique leverages the photovoltaic properties of certain nanostructures, where optical excitation leads to the generation and separation of charge carriers, forming localized electric fields capable of influencing nearby cells. The underlying principle involves creating a photogenerated potential at the nanomaterial–electrolyte interface, thereby modulating excitable cell activity.^[^ ^191^ ^]^ When these interfaces are combined with optogenetic interventions, precise spatial and temporal control offers therapeutic potential for the treatment of spinal cord injury, multiple scleroses, epilepsy, and Alzheimer's disease.^[^ ^192^, ^193^ ^]^

At the most basic level, optoelectronic stimulation can be achieved by forming electrical connections between light‐sensitive NPs, enabling charge transfer across nanoscale junctions. One example of this is the hybrid system developed by Bareket et al.,^[^ ^194^ ^]^ in which CdSe/CdS NRs were conjugated to CNTs. Upon illumination, the photogenerated charges in the NRs are tuned to the carbon nanotubes, resulting in an electric field at the CNT–electrolyte interface. This interface served as the active zone for direct retinal neuron stimulation in vivo. In related work, Chen et al.^[^ ^195^ ^]^ developed self‐assembling zinc porphyrin NRs coated with a TiO_2_ shell, which formed a photoactive core–shell nanostructure. Upon irradiation with single‐ and two‐photon laser sources, rapid electron transfer from the porphyrin core to the TiO_2_ shell occurs, triggering membrane depolarization and the initiation of action potentials in nearby neurons. Notably, in vivo photostimulation of the motor cortex in mice with these NPs elicited motor responses in the hind limbs.

More sophisticated systems enhance the charge separation efficiency by incorporating multilayered optoelectronic structures with staggered energy levels. In such architectures, a photoactive core material is sandwiched between materials that function as electron and hole transport layers. Upon photoexcitation, charges generated within the core are driven in opposite directions by adjacent layers, resulting in charge accumulation at the device surface. A representative example of such a system was reported by Karatum et al.,^[^ ^196^ ^]^ who developed a device featuring a PbS quantum dot layer flanked by ZnO as the electron transport layer and poly(3‐hexylthiophene) (P3HT) as the hole transport layer. When exposed to near‐infrared (780 nm) light, the composite structure generated a capacitive ionic current that successfully triggered action potentials in cultured hippocampal neurons.

### 3.2 Challenges and Future Directions

The complexity of biological environments presents considerable challenges for the use of optically responsive materials. Variable diffusion kinetics, nonspecific interactions eliciting unwanted biological responses, and the detectability of optically active materials inside turbid samples constitute significant challenges for bioengineered and natural samples. The interactions between nanomaterials and biological systems are known to depend on several properties, including size, shape, chemical functionality, surface charge, and composition.^[^ ^197^ ^]^ While numerous currently approved nanotherapeutics demonstrate fewer side effects than their small‐molecule counterparts do,^[^ ^198^, ^199^ ^]^ other NPs, such as quantum dots that may leach cadmium and selenium ions, have been observed to be toxic.^[^ ^200^ ^]^ In addition, excessive light exposure can lead to a range of photothermal, photomechanical, and photochemical toxicity effects. The subject of phototoxicity has been reviewed in detail elsewhere.^[^ ^201^, ^202^ ^]^ In brief, the potential of a certain light stimulus to induce phototoxic damage mainly depends on the energy delivered, which is a function of its intensity and wavelength. The highest energies are generally produced by lasers, so these sources need particular care, together with shorter‐wavelength UV and blue LEDs. Acceptable levels of light exposure to the eye and skin have been agreed upon, and these guidelines have been widely incorporated into national legislation.^[^ ^203^ ^]^

Given the improved contrast and deeper tissue penetration that can be obtained in the 2^nd^ NIR window, there is a clear need for ongoing development of novel materials that exploit this wavelength range. In addition to extending the performance of inorganic materials such as single‐walled carbon nanotubes and Er‐doped NPs, there is a need for genetically encoded fluorescent proteins with much longer emission wavelengths than currently available fluorescent proteins. Similarly, further improvements in optical clearing technologies could improve the contrast and tissue penetration of visible wavelengths. Recently, Ou et al.^[^ ^204^ ^]^ demonstrated that the introduction of highly absorbing molecules could counterintuitively improve optical transparency in live animals.

While photoactivated drug delivery systems have received a great deal of attention (see Section 3.1.2), light‐guided micro/nanovehicles are rapidly emerging as actively guided, on‐demand delivery systems to transport cells, drugs, and biomolecules to targeted tissues. While magnetic fields, electrical fields, and ultrasound have typically been used to propel nanovehicles,^[^ ^205^ ^]^ light can provide additional degrees of freedom to manipulate light‐responsive materials with high spatial and temporal precision (see Figure 8). However, biocompatibility can be challenging for many current nanovehicles that are propelled by asymmetric surface chemical reactions. Alternatively, photothermal effects and thermophoresis can be used to generate a local temperature gradient to propel microstructures that have two or more distinct physical components. Recently, a fast‐moving light‐propelled biodegradable polymeric nanomotor was reported^[^ ^206^ ^]^ on the basis of the nonuniform distribution of Au NPs on the outer surface of bowl‐shaped polymersomes. The significant biomedical potential of these particles was demonstrated by the intracellular delivery of encapsulated doxorubicin as a model hydrophobic therapeutic agent to living HeLa cells, as well as the increased uptake of bovine serum albumin and small interfering RNA following disruption of the cell membrane.

The spatial precision of optical stimuli comes with inherent limitations in tissue penetration depth, particularly for shorter wavelengths that are readily absorbed or scattered by biological tissues. This constraint led us to explore magnetic stimuli, which offer superior tissue penetration capabilities while maintaining noninvasive delivery, in the next section. Magnetic‐responsive materials leverage the unique properties of magnetic fields to achieve deep tissue targeting without the depth limitations that characterize optical approaches.

## 4 Magnetic Responsive

Iron oxide nanoparticles (IONPs) are routinely used in multimodal imaging via both magnetic resonance imaging (MRI) and magnetic particle imaging (MPI). Independently, IONPs of ferromagnetic and ferrimagnetic materials have interesting magnetic properties depending on the particle size, shape, and surface modification. They possess unique characteristics, including chemical stability, biocompatibility, tunability, and, most importantly, strong magnetic properties, making them suitable for a broad range of imaging applications. The collective contributions of IONP size, crystalline morphology, and polydispersity dictate its measured magnetic response and pharmacokinetics. The ability to integrate IONPs as magnetic cores into extended matrix materials (i.e., organic and inorganic polymers) endows them with additional magnetic properties and functional properties. This thereby extends their capabilities from only imaging to respond to exogenous stimuli, expanding their utility to various therapeutic applications, described as magnetic responsive nanocomposites.

Magnetic responsive nanocomposites are a broad class of advanced nanomaterials that undergo significant changes in their behavior or structure when exposed to an external stimulus such as a magnetic field, light, or a change in environment. These materials can be designed to respond dynamically and immediately to varying external forces, enabling controlled manipulation and making them highly versatile in a range of biomedical applications, including their use in imaging, cell tracking, and on‐demand drug delivery.^[^ ^207^ ^]^ The ability to precisely control their response to stimuli offers vast promise in the potential of developing adaptive, on‐demand technologies, particularly in difficult‐to‐reach areas that require remote or real‐time control.

In this section, we explore the modification of IONPs with controllable properties that have emerged as versatile tools in a wide range of biomedical applications, including imaging, drug delivery, hyperthermia therapy, and biosensing.

### 4.1 Physical Properties

#### 4.1.1 Size and Shape

A key determinant of their functionality and performance is particle size, which influences their magnetic properties, biodistribution, cellular uptake, circulation time, and clearance pathways. The relationship between IONP size and its impact on application‐specific outcomes aims to define optimal size ranges for different biomedical uses. For example, smaller IONPs (<10 nm) offer rapid renal clearance, making them ideal for diagnostic applications requiring fast elimination, whereas intermediate sizes (10–50 nm) are typically favored for cellular uptake and MRI contrast enhancement. Larger IONPs (>50 nm) may be better suited for magnetic hyperthermia because of their enhanced heating efficiency. Understanding and tailoring the IONP size to match the requirements of specific applications is critical for maximizing efficacy, minimizing toxicity, and accelerating clinical translation.^[^ ^208^, ^209^ ^]^ For example, mathematical modeling validated by experimental tests has shown that optimal MPI performance is achieved by monodisperse magnetic core solutions of the same size, with the ideal core size being approximately 15 nm in diameter; generally, most reported particles are between 5 and 25 nm in size.^[^ ^210^, ^211^, ^212^ ^]^ In polydisperse settings, the MPI signal is dominated by particles with the optimal core size, whereas off‐sized particles do not contribute to the relaxation time, thereby reducing the sensitivity of the solution. It is also important to consider the magnetic interaction between individual IONP cores. Theoretically, each IONP behaves independently. However, experimentally, changes in medium viscosity and temperature can increase interparticle interactions and cause aggregation.^[^ ^210^, ^212^, ^213^ ^]^

Each disease setting, however, requires IONPs of a different size for optimal uptake, given the difference in disease pathophysiology. For example, particles >80 nm are rapidly cleared by the reticuloendothelial system (RES) and are therefore effective for liver lesion imaging but are not adequate for other tumor imaging applications owing to limited uptake.^[^ ^214^ ^]^ Within oncology, smaller‐sized particles are preferred to enable deeper penetration of the IONP within the tumor. The accumulation of NPs within tumor tissues often relies on the extravasation of IONPs via leaky tumor vasculature. This phenomenon, termed the “enhanced permeability and retention” (EPR) effect, enables passive disease targeting and is generally optimized with smaller nanoparticles in the nanometer range (<25 nm).^[^ ^215^, ^216^, ^217^ ^]^

The capacity for cellular labeling is also dependent on the IONP size. Cell tracking via IONPs is a powerful molecular imaging tool used in a plethora of research areas, including visualizing cell homing mechanisms, monitoring stem cell therapy integration, and understanding cancer metastasis.^[^ ^218^ ^]^ The labeling efficiency, which refers to how much iron can be loaded per cell, greatly differs between IONPs and other cell types, with no ideal methodology. The consensus, however, is that particles with a small core size (25–100 nm range) are more ideal for passive cellular uptake through endocytosis, and phagocytic cells are notably capable of greater loading capacity. As the desity (Dh) of IONPs increases, cell membranes need to be permeabilized with agents such as heparin to facilitate uptake.^[^ ^219^, ^220^ ^]^

Overall, the combination of the physical properties of IONPs influences their imaging performance. It is important to produce well‐defined, homogenous IONP solutions to ensure that the strongest signal is generated. IONPs should also be fine‐tuned for different disease settings to enhance circulation and uptake by the tissue of interest. There are a comprehensive number of reported IONP synthesis methods. However, the method chosen must carefully balance optimizing the performance of IONPs in vivo with their magnetic relaxation behavior for various applications, e.g., imaging or theranostic applications.^[^ ^221^, ^222^ ^]^

#### 4.1.2 Surface Modifications

Modification of IONPs with surface coatings is a common method to impart them with prolonged circulation times and protection against immune clearance but, more importantly, responsive properties. The fate of IONPs is a well‐defined process, with rapid opsonization by phagocytic cells through the mononuclear phagocytic system (MPS), followed by clearance through Kupffer cells of the hepatic system.^[^ ^223^ ^]^ The rapid clearance of naked IOPNs can limit their imaging and therapeutic efficacy, as there is not sufficient delivery to tissues of interest. To ensure that the IONPs circulate for a sufficient length of time, organic and inorganic materials are used as external coatings. The addition of these coatings alters their circulatory behavior and can even change their clearance mechanism. Additionally, they can help alleviate the agglomeration of magnetic cores by inhibiting individual IONP interactions and increasing solution polydispersity.^[^ ^210^ ^]^ The modification of IONPs with reactive functional groups further enables the conjugation of additional targeting moieties or drugs for a theranostic approach.^[^ ^224^, ^225^, ^226^ ^]^ Overall, surface modifications are important steps to modify IONPs into stimuli‐responsive nanoparticles and make them structurally dynamic. In this section, we explore each of these coatings and their use as magnetic responsive materials.

##### Polymers

Natural and synthetic polymers are long repeating chains of different lengths and sizes, representing the most popular IONP coatings used. Polysaccharides, such as dextran and chitosan, provide IONPs with polydispersity and stability, preventing their aggregation in solution. Several studies have shown considerable aggregation of naked IONPs when they are added to water or Dulbecco's Modified Eagles Medium (DMEM), whereas dextran, aminodextran, and carboxymethyl‐dextran substantially less aggregation.^[^ ^212^, ^227^ ^]^ Carboxydextran, a very commonly used coating, also facilitates increased phagocytic cellular uptake, which benefits cellular labeling but is a disadvantage for in vivo applications because of its rapid clearance.^[^ ^219^ ^]^ Synthetic polymer coatings, such as polyethylene glycol (PEG), polyvinylpyrrolidone (PVP), polyvinyl alcohol (PVA), poly(lactid‐co‐glycolid) (PLGA), and poly(maleic anhydride‐alt‐1‐octadecene) (PMAO), endow IONPs with a prolonged half‐life, immune evasion ability, and superior colloidal stability.^[^ ^30^, ^213^, ^228^, ^229^, ^230^ ^]^ PEG is a particularly popular polymer coating and is readily used as a protective coating for many clinically approved molecules.^[^ ^231^ ^]^ PEG shields antigenic sites on the nanoparticle, preventing its detection by the immune system and subsequent sequestration. Polymer coatings also facilitate the addition of functional groups on the IONP surface, such as carboxyl groups, for the attachment of additional structures. This approach is routinely used for the design of cancer theranostic agents by conjugating drug payloads to the IONP, creating a single molecule that detects and treats tumors.^[^ ^224^, ^225^ ^]^ Surface coatings also facilitate multimodal imaging, such as MRI/fluorescence imaging through the attachment of fluorescent agents, MRI/(positron emission tomography) PET imaging through radiolabeling, or MRI/photoacoustic imaging (PAI) through the attachment of other metals.^[^ ^217^, ^232^, ^233^ ^]^

Synthetic and natural polymer coatings clearly differ in their benefits. Keselman et al.,^[^ ^234^ ^]^ for example, compared the circulation of comparatively similar IONPs, one PEG‐coated and the other carboxydextran‐coated. This study elucidated the impressive circulation time of PEG in IONPs, which circulate for more than one hour in the heart, in contrast to Vivotrax, which clear into the liver within minutes. Overall, polymer coatings are crucial for the successful in vivo application of IONPs, as they provide prolonged circulation, colloidal stability and surface properties for further functionalization. Given their popularity and routine clinical use, a variety of synthesis methods are available, and they are generally inexpensive and well tolerated.

##### Metals

Metal doping of IONPs has more recently emerged to improve the response of IONPs to magnetic fields and for multimodal applications.^[^ ^235^, ^236^, ^237^ ^]^ This is commonly achieved by replacing one iron (Fe^2+^) ion with a different metal, including gold (Au), silver (Ag), zinc (Zn), manganese (Mn) or gadolinium (Gd).^[^ ^235^, ^238^, ^239^, ^240^, ^241^, ^242^ ^]^ Modifying IONPs in the context of imaging with metals has the ability to increase their response to magnetic fields, thereby altering their MRI contrast and improving their imaging capabilities. Multiple reports have shown the modification of IONPs with the noble metals Au and Ag due to their stable chemical properties, although many in vivo applications are challenging to find, limiting any conclusions regarding their ability to enhance MRI.^[^ ^240^, ^243^, ^244^ ^]^

Metallic coatings can also impart IONPs with an enhanced response to light stimuli. Several studies have demonstrated the use of dual‐imaging strategies that combine MRI and photoacoustic imaging (PAI).^[^ ^233^, ^245^, ^246^ ^]^ A secondary function of Au coatings is their production of heat when excited by a light‐emitting diode (LED) for PTT of cancer, presenting a theranostic approach.^[^ ^247^ ^]^ Li et al.^[^ ^248^ ^]^ reported a gold‐coated IONP loaded with a chemical drug as a multifunctional theranostic platform. This nanoparticle exhibited distinct functionalities, including molecular MRI using the IONP core for imaging, fluorescence imaging enabled by the intrinsic properties of the chemical drug, and dual therapy combining gold‐mediated photothermal therapy with chemotherapy. These systems highlight the potential of metal‐doped IONPs in integrating diagnostic imaging with targeted therapeutic approaches for enhanced cancer treatment.

##### Lipids, Fatty Acids, and Amino Acids

Amino acids (AAs) and fatty acids (FAs) are commonly used as organic‐based surfactants. As inexpensive, biodegradable, biocompatible, and highly soluble coatings, AA, such as albumin, and FA, such as oleic acid, are widely used in drug delivery and nanoparticle modification. They provide a beneficial precursor base for the attachment of various functional groups and additional coatings, as evident from many of the previously reported IONPs.^[^ ^30^, ^212^, ^225^, ^249^ ^]^ The use of a biodegradable alternative to synthetic polymers for coating IONPs with various poly‐amino acids and oleic acid has resulted in excellent stability over a range of pH values and salt concentrations and increased *R* ^2^ values on phantom scans.^[^ ^250^ ^]^ Similarly, albumin‐coated nanoparticles result in stable particles that cause substantial uptake by cells with low cytotoxicity.^[^ ^251^ ^]^

In addition to AA and FA, lipids are another interesting choice for IONP modification. Magneto‐liposomes (MLs), first introduced in the 1980s, are bilayered phospholipid nanoparticles loaded with IONPs in their hollow center and are an interesting area of nanoparticle research, providing a multitude of benefits to IONP function.^[^ ^252^ ^]^ This includes the inhibition of IONP aggregation caused by protein coronas and protection from their degradation and clearance, therefore positively influencing in vivo behavior. Liposomal coatings can also respond to changes in pH and disintegrate as an on‐target drug release mechanism, which is particularly useful in oncological settings where tumors are more acidic than the surrounding environment.^[^ ^253^ ^]^

### 4.2 Biomedical Applications

With an understanding of the modifications possible for IONPs, the following section explores the resulting applications and the enhanced responsive behaviors conferred by these coatings.

#### 4.2.1 Diagnostic Imaging

MRI is a noninvasive imaging technique that uses strong magnetic fields and radiofrequency pulses to generate detailed three‐dimensional images of internal structures. It is particularly effective in visualizing soft tissues and diagnosing conditions in neurology, oncology, and cardiology, which are widely used in clinical settings.^[^ ^254^, ^255^, ^256^ ^]^ The integration of magnetic‐responsive materials has significantly enhanced the diagnostic capabilities of MRI.^[^ ^257^, ^258^ ^]^ There is a clear paradigm shift toward molecular imaging through the noninvasive detection of underlying pathological biomarkers and away from simple monitoring of anatomical changes and symptom manifestations. The development of biocompatible and highly responsive IONPs can help improve image clarity by altering the magnetic properties of surrounding tissues, increasing the contrast and specificity of the scans. These advancements address clinical needs for more precise diagnostics and expand the applications of MRI in personalized medicine.^[^ ^235^ ^]^

MPI is an emerging noninvasive imaging modality that leverages the unique magnetic properties of superparamagnetic iron oxide nanoparticles to achieve high sensitivity, spatial resolution, and real‐time imaging capabilities.^[^ ^259^ ^]^ Unlike traditional imaging techniques, MPI directly detects SPION without ionizing radiation, enabling quantitative 3D imaging with exceptional contrast and safety. The use of SPION, a highly magnetic responsive material, is central to MPI, as these nanoparticles generate a detectable signal in response to applied magnetic fields. Advances in nanoparticle design, including the optimization of magnetic core size and biocompatibility, have significantly enhanced MPI performance.^[^ ^260^, ^261^ ^]^ Advances in MPI technology have enabled precise tracking and quantification of biological processes, making it a promising tool for applications in vascular imaging, cancer detection, and targeted diagnostics.^[^ ^74^, ^228^, ^262^, ^263^, ^264^, ^265^, ^266^, ^267^ ^]^ MPI can also serve as a synergistic tool alongside hyperthermia therapy, enabling precise localization, real‐time monitoring, and targeted heat delivery to increase therapeutic efficacy.^[^ ^268^, ^269^ ^]^

#### 4.2.2 Locomotion

Magnetic‐responsive nanocomposites offer precise control over locomotion and structural reconfiguration of IONPs into polymer matrices, which enables their response to external thermal and magnetic field stimulation. These systems can be used for targeted drug delivery or hyperthermia therapy, allowing noninvasive navigation to specific locations within the body. Under a rotating magnetic field (RMF), IONPs can transition from random motion in solution to aligned magnetization, forming chains that can be manipulated directionally. This mechanism enables the transport of drug‐loaded nanoparticles through narrow lumens or complex internal anatomy. For example, Ahmad et al.^[^ ^270^ ^]^ demonstrated that low‐frequency RMFs can guide IONP chains toward a target site, such as a tumor, where an external magnetic field can further localize them for specific delivery, minimizing side effects caused by off‐target delivery. Additionally, modifying the shape and structure of the polymer matrix surrounding the IONPs allows for enhanced locomotion and drug release. Tubular shapes are particularly advantageous during transit because of reduced drag forces and limited interactions with surrounding fluids, whereas rectangular conformations at the target site increase the surface area for enhanced drug release.^[^ ^271^ ^]^ Adaptable polymers, such as poly(N‐isopropylacrylamide) (PNIPAM) or polydimethylsiloxane (PDMS), further expand functionality by their ability to change structure, enabling rolling, gripping, or helical motion under magnetic fields. This adaptability is beneficial not only for targeted drug delivery but also for applications in soft robotics, internal sensors, and surgical implants.^[^ ^272^ ^]^ Together, these features make magnetic nanocomposites promising platforms for precision medicine, combining controlled locomotion with multifunctional therapeutic capabilities.

#### 4.2.3 Heat as a Therapy and Release Mechanism

Hyperthermia through the heating of IONPs has long been proposed as a cancer treatment method, but recent advancements have expanded its applications and mechanisms. Heat generation by IONPs can be achieved either through exposure to alternating magnetic fields (AMFs) at varying frequencies or external light stimuli, with the AMF directly interacting with the IONP core and light stimuli targeting surface coatings such as metals, polymers, or hydrogels.^[^ ^273^, ^274^, ^275^ ^]^ This localized heating can damage surrounding tumor tissue directly or trigger nanoparticle disintegration, leading to the release of therapeutic cargo.^[^ ^276^ ^]^ The ability to precisely control heat generation makes this approach highly targeted, as heating is confined to the location of the IONPs and only magnetic materials respond to AMF stimulation. Magnetic hyperthermia therapy stands out as a minimally invasive treatment that uses localized heat to destroy cancer cells while sparing healthy tissue. By increasing the tumor temperature to between 40 °C and 45 °C, cellular and vascular changes are induced that are toxic to both cancer cells and their blood supply. Tay et al.^[^ ^277^, ^278^ ^]^ demonstrated a theranostic platform combining magnetic particle imaging (MPI) with magnetic hyperthermia for image‐guided cancer therapy, achieving precision treatment by confining thermal damage to targeted tumors without affecting healthy organs such as the liver. Additionally, Savari's study highlighted the selective killing of glioblastoma multiforme (GBM) cells via magnetic‐responsive nanocarriers with enhanced efficacy from successive surface coatings and vibration‐inducing AMF, reducing the duration of treatment from 72 h to just 30 s.^[^ ^279^ ^]^ Furthermore, heat‐triggered drug delivery via magnetic stimulation enables precise localization and controlled therapeutic release at target sites, such as during chemotherapy. This system can be used in synergy with hyperthermia, enhancing treatment specificity while minimizing off‐target effects. For a comprehensive review on this topic, Zhang et al. and Mi provided excellent insights into the use of IONPs in hyperthermia therapy for cancer.^[^ ^280^, ^281^ ^]^

In addition to magnetic hyperthermia, IONPs possess strong photothermal conversion efficiency and can therefore be used in PTT, where near‐infrared light induces targeted heat generation. PDT is another minimally invasive modality that uses light, chemical photosensitizers (PSs), and molecular oxygen to produce cytotoxic reactive oxygen species (ROS), selectively destroying tumor cells.^[^ ^282^, ^283^ ^]^ These advanced technologies allow for precise heat‐triggered drug release and maximize the adjuvant effects of hyperthermia in enhancing chemotherapeutic responses. Surface coatings such as metals further improve IONP responsiveness and susceptibility to PTT and PDT applications.

Iron carbide nanoparticles (Fe_3_C NPs) represent a particularly powerful platform for thermal therapy because of their unique magnetic and thermal properties. Compared with traditional IONPs, iron carbide NPs exhibit superior magnetic loss and thermal efficiency and precise heat generation at lower NP doses. These properties not only improve therapeutic outcomes but also facilitate integration with imaging and drug delivery systems for next‐generation theranostics.^[^ ^284^, ^285^, ^286^ ^]^

### 4.3 Other Applications

**Figure 11:**
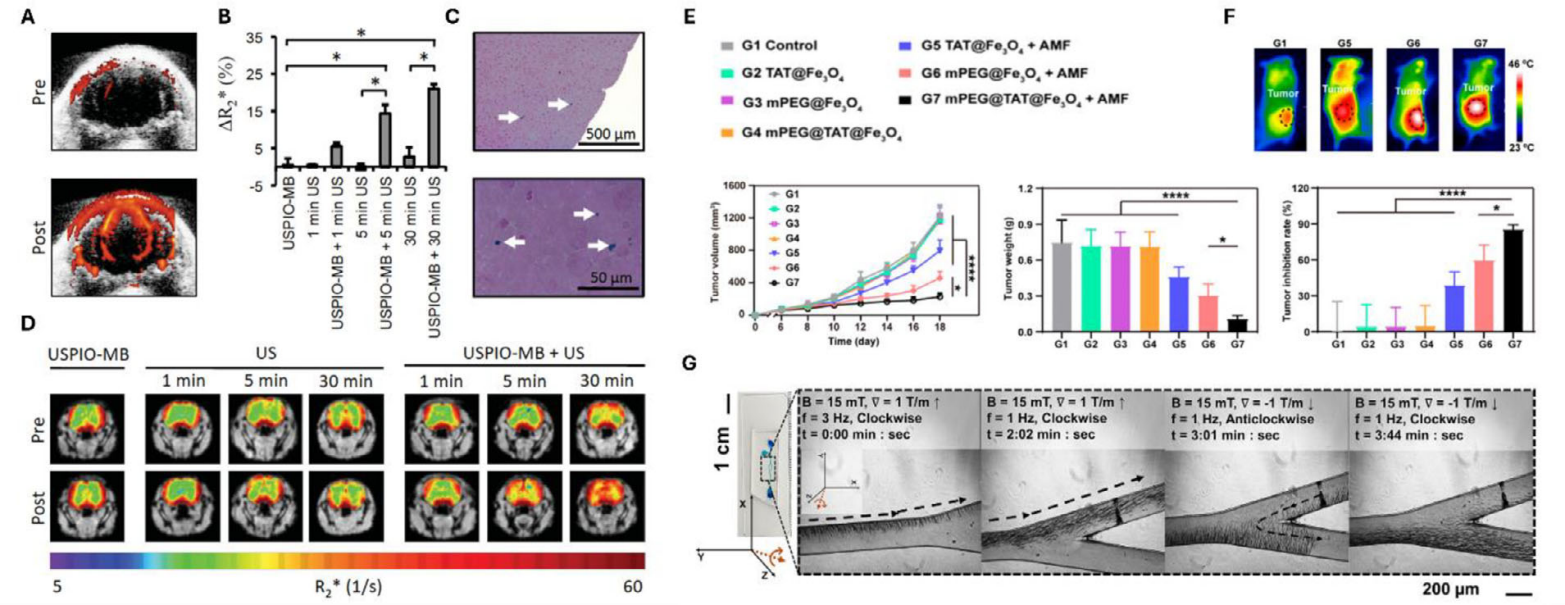
Applications of magnetic responsive materials. A) Monitoring BBB permeation via ultrasound after intravenous infusion of ultrasmall superparamagnetic iron oxide nanoparticles. B) Magnetic resonance imaging quantification of R2* measurements across the BBB after ultrasound‐induced destruction. C) Prussian blue staining of brain tissue confirming the deposition of USPIO nanoparticles. D) Color‐coded R2* magnetic resonance imaging maps. Reproduced with permission.^[^ ^288^ ^]^ Copyright 2015 Wiley. E) CT26 tumor growth curve, tumor weight, and tumor inhibition rate of the mice treated with mPEG@TAT@Fe_3_O_4_. F) Infrared radiation thermal images of CT26 tumor‐bearing mice after the treatments. Reproduced with permission.^[^ ^275^ ^]^ Copyright 2022, ACS Publications. G) Time‐lapse images showing active guidance of the SPIONs in a microfluidic channel with a rotating magnetic field (RMF). The SPION chains were guided into the upper channel by an upward gradient, returned, and guided into the lower channel by a downward gradient. Reproduced with permission.^[^ ^270^ ^]^ Copyright 2022 Wiley.

**Figure** **11** presents data from several applications that utilize the power of magnetic responsive materials and the application of stimulation. While hyperthermia for targeting cancer cells has been well researched, its potential for opening the blood–brain barrier (BBB) presents a distinct challenge. More than 98% of small molecules cannot cross the BBB; however, numerous studies have demonstrated that magnetic hyperthermia through the heating of IONPs at a low radiofrequency can locally and selectively increase BBB permeability without affecting surrounding brain tissue.^[^ ^287^ ^]^ In addition, T2‐weighted MRI of IONPs can be used to assess the extent of BBB disruption. Moreover, Lammers et al demonstrated that the BBB could be effectively opened by delivering ultrasmall IONPs to microbubbles destroyed via transcranial ultrasound pulses.^[^ ^288^ ^]^

Transcranial magnetic stimulation (TMS) is widely recognized as a noninvasive brain stimulation technique that uses magnetic fields to induce electric currents in specific cortical regions, offering therapeutic benefits for various neurological and psychiatric conditions.^[^ ^289^, ^290^ ^]^ Recent advancements have explored the integration of IONPs with TMS to increase treatment precision, efficacy, and neuromodulatory depth. For example, Lu et al.^[^ ^291^ ^]^ investigated the use of IONPs injected into the left prelimbic cortex of mice subjected to chronic unpredictable mild stress (CUMS). This was followed by magnetic stimulation with a 0.1 T field, where the mice demonstrated rapid improvement in their depressive‐like symptoms. This highlights the improvement in treatment efficacy when TMS is combined with IONPs and the ability to target specific neural circuits more effectively, paving the way for innovative approaches to treating mood disorders and other brain‐related conditions.

### 4.4 Challenges and Future Directions

Despite promising preclinical results, magnetic‐responsive materials face significant challenges for clinical translation. Key obstacles include cytotoxicity, immune system interactions (particularly with PEG coatings triggering hypersensitivity reactions), and nanoparticle aggregation, which reduces stability. The surface modification strategies must balance preventing aggregation while maintaining responsive properties. Additionally, scalable production methods must consistently maintain particle size and magnetic properties, and long‐term safety concerns related to oxidative stress remain unresolved. Future directions focus on developing multifunctional platforms that combine therapeutic, diagnostic, and reporting capabilities for personalized medicine. Improving biocompatibility and biodegradability is essential, particularly for engineering surface chemistry to avoid immune clearance and reduce off‐target accumulation. Advancements in external magnetic field systems will enhance spatial and temporal control in vivo. The field is expanding beyond hyperthermia to include mechanical stimulation, controlled drug release, and blood‒brain barrier modulation. Combining magnetic responsiveness with other stimuli (pH, light, and ultrasound) may create sophisticated multistimuli‐responsive platforms with context‐dependent behaviors.

## 5 Ultrasound‐Responsive

While the previous section demonstrated that magnetic stimuli provide excellent tissue penetration and noninvasive control, they still require specialized materials containing magnetic components and often demand high‐power equipment for effective field generation. Ultrasound stimuli present an alternative approach that combines deep tissue penetration with established clinical safety profiles and widely available equipment. Sound waves offer mechanical energy transfer that can be precisely focused and controlled, opening different therapeutic possibilities through acoustic rather than electromagnetic mechanisms.

Ultrasound‐responsive materials represent an innovative class of smart materials that undergo physical or chemical changes when exposed to ultrasonic waves. These materials have gained significant attention in biomedical applications, particularly as ultrasound contrast agents. As contrast agents, they enhance the acoustic reflectivity of target tissues, improving imaging resolution and diagnostic accuracy in various clinical scenarios. Microbubbles, liposomes, and polymeric nanoparticles are common examples, with gas‐filled microbubbles being the most widely used owing to their high echogenicity and responsiveness to ultrasound. Beyond imaging, these materials serve crucial therapeutic functions through mechanisms such as sonoporation, where ultrasound temporarily increases cell membrane permeability, enabling increased drug delivery to targeted tissues. They can also function as drug carriers that release therapeutic payloads precisely when triggered by ultrasound at specific sites, minimizing systemic side effects. Additionally, some formulations enable sonodynamic therapy, where ultrasound activates sonosensitizers to generate reactive oxygen species for tumor destruction, and others facilitate high‐intensity focused ultrasound treatments for noninvasive thermal ablation of pathological tissues.

### 5.1 Principles of Ultrasound Contrast Agents

Microbubbles (MBs) have been used as ultrasound contrast agents (UCAs) in clinical practice for several decades.^[^ ^34^, ^292^ ^]^ Their first use was half a century ago when the use of ultrasound for medical diagnostics was in its infancy,^[^ ^293^ ^]^ and the safe use of UCAs has been endorsed by multiple national and supranational clinical societies.^[^ ^294^ ^]^ However, they are still not as commonly used as other medical‐imaging agents. They are clearly a material that responds to the stimulus of sound waves, but their mechanism of operation is not always understood by practitioners; a better understanding may lead to better usage.

Unlike, for example, elements such as iodine, which provide X‐ray contrast, or gadolinium, which provides magnetic resonance contrast, MBs do not affect imaging radiation at the atomic level. This is because the stimulus of sound waves is a mechanical oscillation affecting materials in the bulk rather than at the atomic or molecular level; thus, an agent that responds to sound must be a mechanical oscillator. Gases are much more compressible (or “springy”) than liquids are, while liquids may be almost a thousand times denser than gases are. Thus, a gas bubble surrounded by a liquid may be thought of as a type of spherical “spring” attached to the mass of liquid around it. If a bubble is temporarily compressed (“squeezed”) and then released, it will bounce outward, pushing the mass of liquid outward. This motion typically overshoots, causing the gas in the bubble to expand (“stretched”), causing it to bounce back inward, and so on, “ring” in response to any initial “squeeze” or “stretch”. The details of this physics were understood by the early 20th century^[^ ^295^, ^296^ ^]^ and have been mathematically explained in a number of books,^[^ ^297^, ^298^, ^299^ ^]^ with excellent predictions of the observed behavior.

Most significantly, similar to a mass bouncing on a spring, there is a natural frequency with which the bubble oscillates. The bubble's volume, not its shape, oscillates. The natural frequency is inversely proportional to the radius of the bubble; for an air bubble in water (or blood) near atmospheric pressure, the constant of proportionality is approximately 3.3 m ^−1^s and hardly differs from this value for other gases. Hence, a bubble that is 1 mm in radius, which one might see on pouring water into a glass, “rings” at ≈3.3/0.001 or 3300 Hz, a frequency we hear and associate with water in motion. Moreover, a bubble that is 1 micron in radius “rings” at ≈3.3 MHz is well within the range of medical ultrasound scanners (although materials of the microbubble “shell” do shift this natural frequency).

When a bubble is in a sound field, such as that of an ultrasound scanner, the high‐pressure “crests” of the sound waves compress the bubble, whereas the low‐pressure “troughs” of the sound waves expand the bubble. If the sound frequency is similar to the bubble's natural (“ringing”) frequency, the result is resonance: the bubble expands greatly and contracts greatly as each wave passes. The aforementioned constant of proportionality of approximately 3 m s^−1^ defines the size of the bubble that resonates at a given sound frequency, but the ≈1500 m s^−1^ of the speed of sound in water‐like liquids or human tissues defines the minimum size of an object that can be imaged at a given frequency: orders of magnitude larger than the bubble. The consequence is that an individual bubble is far too small to be seen in an image. However, since the bubble resonates, it strongly absorbs some of the applied sound energy. It simultaneously reradiates much of this absorbed energy, but the reradiation is in the form of spherical sound waves emanating from a source much smaller than the wavelength, waves that thus fall off sharply with distance rather than returning energy to the original planar sound wave. Therefore, a cloud of effectively invisible bubbles can very strongly scatter ultrasound and are interpreted by the scanner algorithms as if they were extremely dense tissue such as bone.

Owing to their convenient and vital coincidence, bubbles that are microns in size are similar in scale to blood cells. Thus, they not only show up strongly on ultrasound scans but also pass through the finest capillaries in the body without causing obstruction. This also means that they can be administered by simple intravenous injection. However, MBs are still too large to pass through the extracellular matrix between endothelial cells, as can many molecular agents. This means that microbubbles are essentially vascular agents that are limited in their ability to detect diseases of the vasculature, diseases featuring abnormal vasculature, or abnormal vascular‐endothelial markers. Research into ultrasound‐responsive nanobubbles that can penetrate vascular endothelia is ongoing.

Microbubble UCAs need not be restricted to blood vessels; they have also been used in other lumens. However, lumens where gases are typically present, such as the gastrointestinal or pulmonary systems, are generally off‐limits to ultrasound imaging owing to the overwhelming reflectivity of gas‒liquid interfaces: unlike MBs, these gas‒filled zones are large enough to directly interfere with ultrasonic waves. The large expansions and contractions due to resonance indicate that the behavior of the bubble is nonlinear, which has been extensively studied.^[^ ^300^, ^301^ ^]^ The nonlinearly oscillating bubble emits harmonics that are different from the original ultrasound frequency, and modern commercial scanners exploit this with a filter that the clinician can switch in, showing only the regions populated with UCAs.

### 5.2 Material Types

The earliest UCAs were uncoated, short‐lived bubbles. The vigorous agitation of a saline solution suitable for intravenous injection and saturation with dissolved air should cause some air to leave the solution in the form of MBs of various sizes. This simple preparation generated the first UCAs to be used in clinical trials.^[^ ^292^ ^]^ However, any bubble has a higher pressure inside than outside, owing to surface tension, a pressure that is inversely proportional to the radius. For MBs, this pressure is high enough to force the air in the bubble to redissolve in the surrounding liquid, so an air bubble several microns in size survives for a matter of seconds^[^ ^302^ ^]^ before harmlessly dissolving in the blood into which it was injected, drastically limiting the distance over which the agent is effective.

Thus, in the last 25 years, UCAs have been made of protein,^[^ ^303^ ^]^ polymer, or lipid^[^ ^304^ ^]^ shells, which improve longevity in vivo, increase echo contrast, and provide a substrate for the attachment of targeting ligands and therapeutic molecules. The manufacture of protein‐shelled agents is relatively simple, and most are made from serum albumin, a very common blood protein with minimal immunogenic risk. Human serum albumin is required for actual clinical use^[^ ^305^ ^]^ and represents the first generation of commercial UCAs.^[^ ^306^ ^]^ Typically, these agents are made by dissolving serum albumin in water that is sonicated by high‐power ultrasound^[^ ^303^ ^]^ with appropriate temperature control. The resulting agents are stable enough to be stored in a conventional refrigerator and have shell thicknesses of 15–50 nm.^[^ ^292^, ^307^, ^308^ ^]^ However, the protein shell is readily ruptured, even by the pressure induced by injection through a fine‐gauge needle,^[^ ^308^ ^]^ and the expansions and contractions of the ultrasonic sound waves cause further stresses, accelerating shell rupture. Once the shell is breached, the released gas rapidly dissolves, and thus, the agent does not last long in the ultrasound beam before disappearing. Nevertheless, these agents remain of clinical utility, and one brand is still commercially available.^[^ ^292^ ^]^ The gas inside the bubble was also improved by replacing air with perfluorocarbon or with sulphur hexafluoride,^[^ ^309^ ^]^ which dissolves only slowly in the blood.

Lipid‐shelled bubbles have largely superseded protein‐shelled MBs. These are phospholipids, the same class of molecules that form cell membranes. Lipid‐shelled agents are not typically stable enough to survive long‐term storage or transport. Therefore, they are made by providing the lipids in an emulsion in aqueous liquid with the appropriate gas dissolved near saturation. Immediately prior to injection, the practitioner must agitate the vial supplied by the manufacturer; MBs emerge from the solution, and the required lipid shell self‐assembles from the surrounding emulsion. By the early 21st century, there were three main competing brands of lipid‐based UCAs, which continue to be in clinical use.^[^ ^292^ ^]^

### 5.3 Applications

#### 5.3.1 Diagnostic Uses of Ultrasound Contrast Agents

The first regular use of UCAs was in echocardiography, where as noted earlier, saline water was agitated to encourage dissolved air to appear in the form of MBs. The simple intravenous injection of agitated saline provided a brief but helpful improvement in the imaging of the right side of the heart. Commercially available agents with the shells noted above survive long enough, including surviving the pulmonary circulation, to provide contrast in the entire vasculature following intravenous injection and are used in modern cardiac examinations.^[^ ^302^, ^310^ ^]^ In addition to the advantage of an intravenous injection over the much more invasive arterial catheter needed for X‐ray contrast imaging, unique techniques are available owing to the nature of the MBs. In brief, a focused increase in the ultrasound beam intensity can be applied to destroy only the UCAs in a region of interest, such as part of the heart muscle; upon returning the ultrasound intensity to normal imaging levels, perfusion of the muscle can be examined as fresh UCAs enter the region.^[^ ^310^ ^]^

Studies of the liver have shown the significant use of UCAs over the last few decades.^[^ ^311^ ^]^ Agents for other imaging modalities may spread among all liver cells, which specialize in detoxifying the blood, but UCAs remain in the vasculature. The liver may be the site of tumors, and since UCAs excel at revealing the vasculature, the abnormal vasculature characteristic of tumors may be revealed.^[^ ^312^ ^]^ Not all clinical use of UCAs requires intravenous injection. MBs are used in gynecological examinations to assess fallopian tube patency.^[^ ^313^ ^]^

As with many other imaging agents introduced into the human body, an important application is the targeting of these materials to particular disease markers. Targeted UCAs have been investigated extensively since MBs were first produced with shells^[^ ^31^, ^32^ ^]^ but are not yet in routine clinical use. Ligands that are specific to the target disease marker need to be attached to the microbubble shell. The most versatile molecule is an antibody, or immunoglobulin protein, which can be engineered to bind to the target molecule. It may be easier to attach antibodies to proteinaceous shells than to lipid‐based shells, but in either case, care must be taken to ensure that the method of attachment is not itself immunogenic or carcinogenic. Microbubble UCAs have been targeted to vascular markers of inflammation,^[^ ^314^ ^]^ which is relevant to many conditions, including cardiovascular disease, inherently exploiting the vascular nature of UCAs.

Finding cancer cells is more problematic; even though many tumors exhibit abnormal vasculature, as noted above, with targeting, one hopes to identify abnormal lesions before they have grown sufficiently to develop substantial neovasculature. Microscopic malignancies may promote the growth of nearby blood vessels via pathological vascular endothelial growth factor expression, which can be targeted.^[^ ^315^ ^]^ A finite volume, or bolus, of agent is injected intravenously. On the scan, the MBs that have not bound to targets are soon swept through the vasculature, but those that have bound remain adhered, revealing the target.

**Figure 12:**
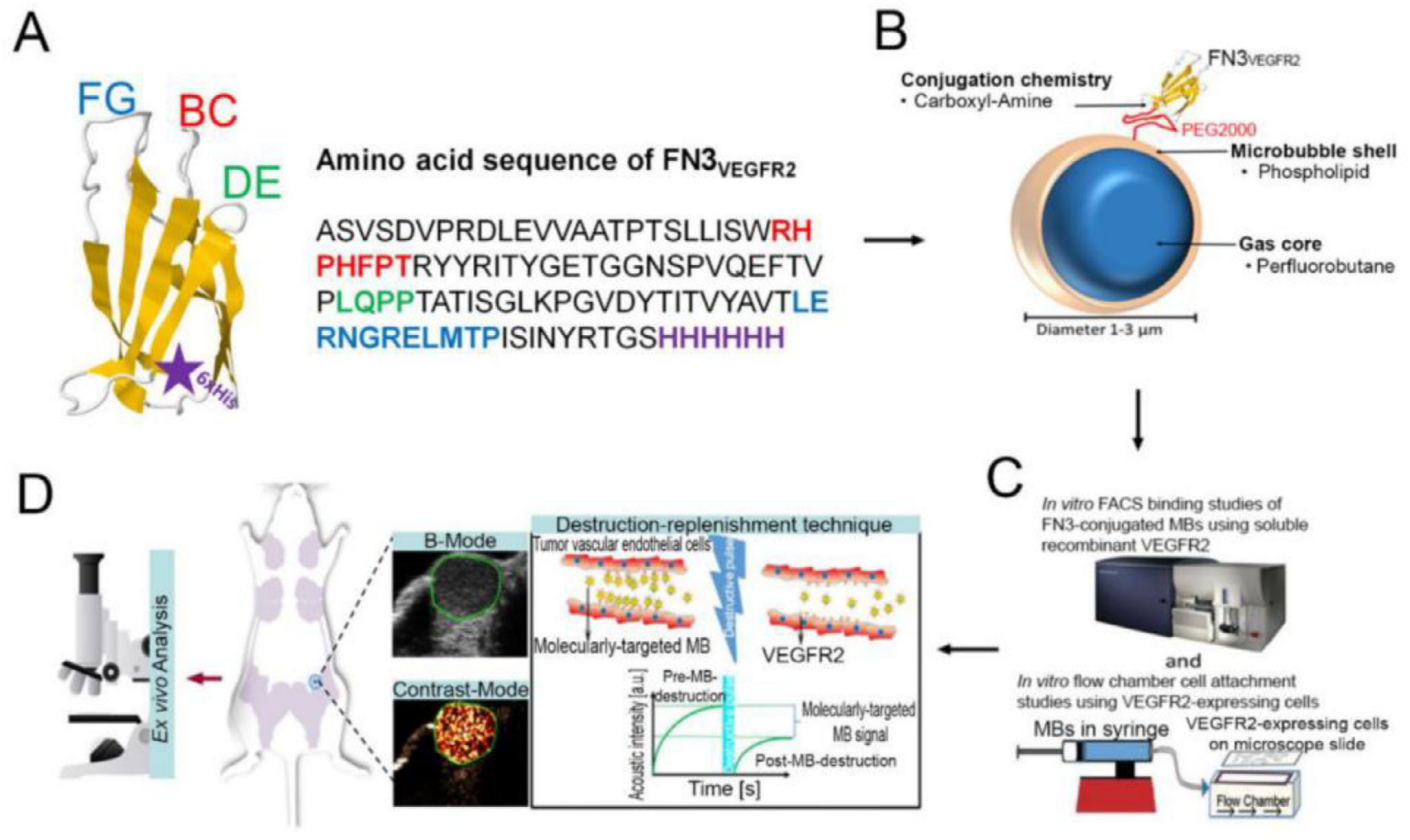
A) Structure of the FN3 scaffold with the corresponding amino acid sequence of the FN3VEGFR2 binder backbone and loop sequences BC (red), DE (green), and FG (blue) with a C‐terminal 6xHis‐tag (purple). Source: protein data bank (http://www.rcsb.org/pdb/home/home.do); search option with ID: 1TTG. B–D) Overview of the overall study design. MB‐FN3VEGFR2 was generated by covalently attaching the FN3 scaffold on the surface of MB. C) Molecularly targeted MBs were tested both in vitro and D, in vivo, in a transgenic mouse model of breast cancer, followed by ex vivo quantitative immunofluorescence of VEGFR2 expression on the tumor neovasculature. The USMI signal was measured via the destruction‒replenishment technique. Reproduced with permission.^[^ ^316^ ^]^ Copyright 2016, Ivyspring International Publisher.

Abou‐Elkacem et al.^[^ ^316^ ^]^ demonstrated that molecularly targeted MBs have great promise as contrast agents for ultrasound‐based cancer imaging (**Figure** **12**). This study evaluated a novel class of potentially clinically translatable MBs via the use of an engineered human‐fibronectin scaffold‐ligand (MB‐FN3VEGFR2) to image VEGFR2, a marker of cancer blood vessel formation. Researchers have assessed binding capabilities through flow cytometry and flow‐chamber cell attachment studies, which demonstrated significantly greater binding to VEGFR2 than control agents did. In vivo ultrasound molecular imaging in a transgenic mouse model of breast cancer revealed specific binding to VEGFR2, which was significantly greater in breast cancer tissue than in normal breast tissue. These findings were confirmed by ex vivo immunofluorescence analysis, which revealed increased VEGFR2 expression in cancerous tissue. These results suggest that MBs coupled to FN3 scaffolds can effectively detect breast cancer neoangiogenesis.

#### 5.3.2 Therapeutic Uses of Ultrasound Contrast Agents

An obvious extension of targeted diagnostic imaging is targeted therapeutics. Once the microbubble has adhered to a target cell, increasing the intensity of the ultrasound beam to rupture the microbubble may induce sufficient local stresses on the cell to kill it. However, constructing a microbubble shell from an appropriate drug while maintaining the targeting properties of the outermost layer of the shell is more reliable. Upon increasing the intensity of the ultrasound beam, the microbubble ruptures, releasing the drug locally.^[^ ^33^, ^317^ ^]^ Should enough MBs be appropriately targeted and ruptured, the drug would be delivered at a high local concentration.^[^ ^34^, ^318^ ^]^ Of course, if the location of the target is known from a prior examination, targeting and drug delivery need not be accomplished with the same agent. Drug‐delivered MBs may be untargeted but rupture when a high‐intensity beam is focused on the target lesion. Cancer treatments using MBs and similar ultrasound‐sensitive injectable agents have been studied extensively.^[^ ^319^, ^320^ ^]^

When the target is a blood clot, the objective is thrombolysis,^[^ ^321^, ^322^ ^]^ a phenomenon discovered serendipitously when the brains of stroke patients are scanned with transcranial ultrasound probes. This application of UCAs has seen significant clinical use.^[^ ^323^ ^]^ Here, the sonothrombolytic effect may be a combination of two phenomena. Mechanical damage may occur from the stresses of rupturing MBs, or gentler local “stirring” may occur due to microstreaming generated by resonating bubbles.^[^ ^324^, ^325^ ^]^ This stirring is thought to effectively mix the thrombolytic drugs typically administered to blood‐clot victims into the clot material without requiring the microbubble shell to be loaded with the drugs.

A particularly interesting therapeutic application of MBs is sonoporation. Here, without the resonating microbubble rupturing, or prior to its eventual rupture, vascular endothelial cells are found to absorb large molecules that they otherwise would not absorb.^[^ ^325^, ^326^, ^327^ ^]^ One of the most promising sonoporation applications is in the brain, where the blood‒brain barrier blocks the passage of many therapeutic molecules that can easily pass from the blood to the underlying tissues elsewhere in the body. Microbubble‐mediated sonoporation has been demonstrated in the brains of both animals^[^ ^328^, ^329^ ^]^ and humans.^[^ ^330^, ^331^ ^]^ Most importantly, the sonoporative effect appears reversible such that normal blood‒brain function resumes after treatment. Microbubble‐mediated sonoporation has also been used for gene transfection.^[^ ^332^, ^333^ ^]^ Again, when cells are persuaded by the highly local mechanical stimuli of resonating MBs to engulf molecules that they otherwise would not, constructing an elaborate microbubble shell containing therapeutic molecules is not necessary. As long as the molecule to be delivered (a gene, in this case) is coincidentally flowing in the vasculature, delivery occurs.

The fundamental physics causing the aforementioned microstreaming phenomenon is a second‐order rectification of the purely oscillating motion of sound waves, producing a net fluid flow, or mean current.^[^ ^299^ ^]^ The to‐and‐fro movements generated by waves are much stronger than the mean currents are, but they cancel out, whereas rectified motions do not. Such currents are common in many systems dominated by mechanical waves; the “rip current” endangering swimmers at surf beaches is a well‐known example. These mean currents only require some change in the symmetry of the system to arise.

When sound waves periodically expand and compress piezoelectric materials,^[^ ^334^ ^]^ the resulting rectification can be a net flow of electrons: a mean electric current. Ultrasound‐generated piezoelectric currents have been proposed for use in therapeutic contexts, including neural stimulation.^[^ ^335^ ^]^ Even without introduced piezoelectric materials, stretch‐activated ion channels in cell membranes respond to steady and oscillatory mechanical stimuli, causing profound changes in cell behavior.^[^ ^336^ ^]^ Moreover, at levels too low to cause mechanical damage, ultrasound causes neuromodulation, which is thought to occur via the stimulation of calcium‐ion channels,^[^ ^337^ ^]^ which may generate a rectified ion current.

Finally, at high enough ultrasonic power, the microbubble not only ruptures but also may briefly expand by an order of magnitude and then collapse, imploding violently.^[^ ^338^ ^]^ Indeed, without any injected MBs, high‐power ultrasound results in cavitation, resulting in the formation of collapsing bubbles.^[^ ^297^, ^299^, ^302^ ^]^ Ultrasonic cavitation is extensively used by commercial devices to clean surfaces, lyse biological cells, and use high‐intensity focused ultrasound (HiFU) to perform scalpel‐free surgical procedures.^[^ ^339^ ^]^ This collapse causes the temperature inside the bubble to rise to thousands of degrees, dissociating the water‐vapor molecules to form hydroxyl radicals.^[^ ^340^ ^]^ These, in turn, diffuse into the surrounding liquid, forming reactive oxygen species (ROS), such as hydrogen peroxide, and promoting chemical reactions that otherwise would not occur; ROS production is among many such reactions collectively termed sonochemistry.^[^ ^341^ ^]^ In addition to the surgical context of HiFU, low‐intensity ultrasound, such as that emitted by a standard medical scanner, can still be arranged to cause controlled cavitation by injecting agents based on liquid nanodroplets coated with chemical agents.^[^ ^342^ ^]^ The droplets vaporize in the ultrasonic beam, forming MBs that then collapse and generate ROS from the molecules of the coating. Ultrasound‐sensitive agents have been shown to have the potential to inhibit bacterial growth via biofilm disruption, in part owing to the generation of ROS.^[^ ^343^ ^]^

### 5.4 Challenges and Future Directions

The vascular nature of ultrasound contrast agents has often been discussed as a limitation, but as noted above, there are applications where this is an advantage. The inability of MBs to penetrate vascular endothelia has led to extensive research into nanobubbles over the last 10–15 years.^[^ ^344^, ^345^ ^]^ Nanobubble materials are small enough to penetrate between endothelial cells, particularly in tumors where vascular endothelia tend to be “leaky”. However, the basic physics still dictates that agents that are smaller require higher frequencies, limiting the depth into the body that ultrasound stimuli can reach. Nanobubbles are by definition less than a micron in size, but the first‐prepared nanobubbles^[^ ^346^ ^]^ are still approximately 0.5 microns in diameter rather than much smaller and hence still resonate at ≈7 MHz; more recent nanobubbles are approximately 0.3 microns in diameter and resonate at ≈12 MHz.^[^ ^345^ ^]^ Nanobubbles have also been produced as gas cores that are only 20–50 nm in size within a 400 nm particle of cyclodextrin, a common drug‐delivery material, and insonated at 18 MHz.^[^ ^347^ ^]^ While these high frequencies do not penetrate as deep into the human body as 1 MHz, they completely insonate a mouse and are well within the range of commercial medical scanners, permitting studies on mouse tumors or atherosclerosis models. Advanced signal processing based on altered physical properties of bubbles adhered to targets may permit nanobubble use with lower‐frequency scans.^[^ ^318^, ^348^ ^]^

To date, ultrasound scans have been highly operator dependent. Unlike X‐ray or MRI, the skill of the practitioner in moving the ultrasound probe over the body while interactively watching the screen has made ultrasound less reproducible than other imaging modalities. The concomitant advantage is that the practitioner is not simply a passive technician but can actively modify the scan to discover unusual features. Future trends may include a robotization of scanning, reducing interpractitioner variability without eliminating practitioner decision‐making.^[^ ^349^ ^]^

## 6 Commercial Translation of Stimuli Responsive Technology

Each stimulus modality, i.e., electrical, optical, magnetic, or ultrasonic, offers distinct advantages and faces unique challenges in terms of tissue interaction, spatial control, and clinical implementation. We now address the critical question of how to systematically select the most appropriate approach for specific clinical needs and navigate the complex pathway from laboratory innovation to clinical reality. This section outlines a framework for selecting the most appropriate SRM and stimulus modality to suit a clinical application as well as the translation (i.e., bench‐to‐bed bedside) challenges faced by these multifaceted materials and technologies.

### 6.1 Framework for Selecting Appropriate Stimuli

Having explored various types of stimuli‐responsive materials, developing a systematic approach for selecting the most appropriate stimulus for specific biomedical applications is crucial. As discussed throughout this review, each stimulus type offers unique advantages in terms of penetration depth, spatial resolution, temporal control, and biological interaction mechanisms. Electrical stimuli provide excellent temporal control and direct interfaces with bioelectrical systems. Optical stimuli provide unparalleled precision and temporal control but are limited by tissue penetration constraints. Magnetic stimuli offer superior tissue penetration and complete noninvasive application. Ultrasound stimuli combine excellent penetration with good spatial resolution and establish safety profiles.

When choosing between different stimuli for biomedical applications, researchers and clinicians must consider several key factors that influence the effectiveness, safety, and practicality of the approach. This framework provides a comparison of electrical, light, magnetic, and ultrasonic stimuli to guide selection on the basis of specific application parameters.

### 6.2 Comparative Analysis of Stimulus Types

This comparative analysis enables systematic evaluation of stimulus modalities by examining multiple parameters simultaneously. When comparing parameters across stimulus types, those most critical to your application, such as tissue penetration for deep targets, spatial resolution for precise interventions, or temporal control for dynamic responses, are prioritized. To assist in interpreting the information provided in **Table** **1**, begin by identifying the critical requirements of an application and then eliminating stimulus types that cannot meet minimum thresholds. Among the remaining options, the parameters most important for therapeutic success were ranked.

**Table 1:** Comparative analysis of stimulus types.

Parameter	Electrical	Light	Magnetic	Ultrasound
**Tissue Penetration** ^a)^	Limited; 1–5 mm surface, up to 2 cm with implanted electrodes	Limited; 0.1–10 mm depending on wavelength: UV 0.1 mm, visible 1–3 mm, NIR 5–10 mm	Good; 5–15 cm depending on field strength, unlimited with implants	Excellent; >20 cm, depth limited mainly by absorption)
**Spatial Resolution** ^b)^	Moderate; 0.1‐1 mm with microelectrodes, 1–5 mm with surface electrodes	Excellent; 0.01‐1 mm with focused beams, diffraction‐limited	Moderate; 1–10 mm depending on coil design and field gradient	Good; 0.5‐2 mm with focused transducers, 5–15 mm with unfocused
**Temporal Control** ^c)^	Excellent microseconds to continuous, frequency range: DC‐100 kHz)	Excellent nanoseconds to continuous, pulsed or CW, frequency range: THz)	Good milliseconds to continuous, frequency range: 1 Hz‐10 MHz)	Good microseconds to continuous, frequency range: 20 kHz‐100 MHz
**Safety Concerns**	Tissue damage >100 V cm^−1^, electrode corrosion, pH changes, current density limits 1–10 mA cm^−^^2^	Phototoxicity >1 J cm^−^^2^, tissue heating >50 °C, retinal damage risk	Minimal with fields <1 Tesla, heating with strong fields >3 Tesla, peripheral nerve stimulation	Thermal effects at intensities >3 W cm^−^^2^, cavitation >1 MPa, standing wave patterns
**Response Specificity**	High with conductive materials (metals, conductive polymers like PEDOT:PSS, PPy)	Highly specific with photoresponsive moieties (azobenzenes, spiropyrans, upconversion nanoparticles)	Specific to magnetic materials (iron oxide nanoparticles, ferrite, rare earth magnets)	Broad response in various materials (microbubbles, sonosensitizers, piezoelectric materials)
**Noninvasiveness**	Often requires implanted electrodes (90% of applications), surface application possible	Noninvasive for surface tissues, fiber optic delivery for deep tissues	Completely noninvasive (100% of applications)	Completely noninvasive (>95% of applications, some require microbubble injection)
**Biocompatibility Rating**	Moderate; for electrode materials require biocompatible coatings, titanium, platinum	High for visible light; Moderate; for UV with potential DNA damage	High for magnetic materials generally inert, FDA‐approved iron oxides	High for ultrasound gel biocompatible, established safety profile
**Energy Requirements**	Low; 1–100 mW typical, up to 10 W for therapeutic applications	Low to Moderate 1 mW‐10 W depending on application	Moderate to High; 100 W‐50 kW depending on field strength	Moderate; 10–500 W for therapeutic applications

^a)^ Tissue penetration ratings indicate the effective therapeutic range: limited (<5 mm), good (5–50 mm), and excellent (>50 mm).

^b)^ Spatial resolution reflects targeting precision: excellent (<1 mm), good (1–5 mm), and moderate (5–20 mm).

^c)^ Temporal control describes response timing: excellent (microseconds to milliseconds), good (milliseconds to seconds).

### 6.3 Biological Considerations

This section provides readers with additional aspects to consider (aligned with Table 1) from a biological perspective when deciding upon the stimulus method and therefore the materials for bioengineering applications.

#### 6.3.1 Target Tissue Accessibility

Surface tissues (0–2 mm depth) are ideal for light‐based stimuli, whereas deep tissues (>5 cm) require magnetic or ultrasound stimuli. Intermediate depths (2–20 mm) offer flexibility in stimulus choice. Penetration depth limitations arise from fundamental physical principles governing energy attenuation in biological tissues. Light‐based stimuli, particularly in the near‐infrared window (700–1000 nm), can effectively reach superficial targets such as epidermal wound healing applications or transcutaneous drug delivery patches. However, tissue scattering and absorption coefficients increase exponentially with depth, limiting effective phototherapy beyond 2–3 mm in most tissue types. For applications requiring deeper penetration, such as spinal cord stimulation or deep brain stimulation, magnetic field gradients or focused ultrasound have become necessary because of their superior tissue penetration properties. The intermediate depth range (2–20 mm) encompasses many clinically relevant targets, including peripheral nerves, subcutaneous drug depots, and superficial muscle groups, where multiple stimulation modalities remain viable options. Selection criteria for this depth range should consider factors such as the required spatial resolution, temporal precision, and specific desired biological response.

#### 6.3.2 Tissue‐Specific Sensitivity

Neural tissue sensitivity to electrical stimulation varies significantly across different cell types and anatomical locations, thus requiring a wide range of stimulation parameters (e.g., stimulus magnitude, duration and waveform).^[^ ^350^ ^]^ In the clinical environment, three different methods are used for bone tissue stimulation: DC,^[^ ^351^ ^]^ pulsed electromagnetic field (PEMF),^[^ ^352^ ^]^ and capacitive coupling (CC).^[^ ^353^ ^]^ Magnetic drug‐targeting systems use iron oxide nanoparticles (10–100 nm) guided by external magnetic fields of 0.1–0.6 Tesla to concentrate therapeutic agents at specific vascular targets.^[^ ^354^, ^355^ ^]^ Recent clinical trials have demonstrated successful magnetic targeting of chemotherapeutic agents to liver tumors, reducing systemic toxicity by 60–80% while maintaining therapeutic efficacy.^[^ ^356^ ^]^

#### 6.3.3 Safety Thresholds

Electrochemical tissue damage mechanisms involve pH changes, gas bubble formation, and toxic metal ion release from electrodes. Therefore, it is imperative that safe chronic stimulation magnitudes and waveforms are employed with many stimulation parameters reported in the literature related to spinal cord injury,^[^ ^357^ ^]^ bone regeneration,^[^ ^358^ ^]^ and wound healing.^[^ ^359^ ^]^ Magnetic field safety considerations extend beyond the static field strength to include gradient switching rates and specific absorption rate (SAR) limits. The FDA guideline of 4 W kg^−1^ whole‐body SAR for MRI procedures translates to approximately 0.5–1.0 Tesla for most therapeutic magnetic stimulation devices.^[^ ^360^ ^]^ Ultrasonic thermal safety margins depend on the exposure duration, with the thermal index (TI) providing real‐time safety monitoring. The mechanical bioeffects of ultrasound become significant above the mechanical index (MI) value of 1.9,^[^ ^361^ ^]^ particularly in gas‐containing tissues. Light‐induced photochemical damage mechanisms vary dramatically across the electromagnetic spectrum. UV‐B radiation (280–315 nm) causes direct DNA damage at doses as low as 10 mJ cm^−^^2^, whereas near‐infrared light (800–1200 nm) can be safely applied at power densities exceeding 100 mW cm^−^^2^ for photobiomodulation therapy.^[^ ^362^ ^]^ Visible light phototoxicity primarily occurs through photosensitizer activation, which requires the consideration of endogenous chromophores such as hemoglobin and melanin.

#### 6.3.4 Stimulation Duration

Acute applications (seconds to minutes) favor electrical or light stimuli. Chronic applications (hours to days) benefit from magnetic or ultrasound stimuli. Temporal considerations reflect both physiological adaptation mechanisms and device engineering constraints. Acute electrical stimulation leverages rapid neural membrane depolarization and immediate cellular responses, making it ideal for applications such as defibrillation (milliseconds), transcutaneous electrical nerve stimulation (minutes), or optogenetic neural control (seconds to minutes). However, chronic electrical stimulation faces challenges such as electrode corrosion, tissue encapsulation, and neural accommodation effects. Conversely, magnetic and ultrasound stimuli can be applied continuously without direct tissue contact, avoiding many chronic implantation complications. Pulsed electromagnetic field therapy for bone healing can be performed continuously for 6–12 h daily over several months, whereas low‐intensity pulsed ultrasound treatments typically require 20‐minute daily sessions for weeks.^[^ ^363^ ^]^ The biological half‐lives of therapeutic responses also influence duration selection: immediate neural responses favor electrical stimuli, whereas slower tissue remodeling processes (collagen synthesis, angiogenesis) align better with sustained magnetic or ultrasound protocols. Recent developments in duty cycle optimization have allowed hybrid approaches that combine acute high‐intensity stimulation with chronic low‐level maintenance protocols.

### 6.4 Technology Readiness Levels and Challenges to Technology Translation

The technology readiness level (TRL) is a systematic measurement scale from 1–9 that assesses the maturity of a particular technology, ranging from basic principles observed (TRL 1) to an actual system proven in an operational environment (TRL 9). The clinical translation of SRM technologies varies significantly across different stimulus modalities, with magnetic‐responsive materials for MRI contrast achieving the highest technology readiness levels (TRL 7–9), demonstrating established clinical utility. Ultrasound‐responsive systems show strong clinical potential (TRL 5–9 for imaging applications), whereas electrical and optical stimuli‐responsive materials remain in earlier development stages (TRL 2–6) (**Table** **2**).

**Table 2:** Examples of SRM for each modality with indicative applicable medical applications, status of application and TRL.

Stimuli Modality	Key Material Class/Examples	Medical Application(s)	Status for this Application	Estimated TRL (General Range/Stage)
**Electrical**	Conductive Polymers, Electrically Responsive Hydrogels, Carbon Nanotubes, Graphene	Neural Interfaces, Cardiac Tissue Engineering Drug Delivery, Biosensing	Direct cell stimulation, tunable properties, high sensitivity for sensors	TRL 2–6 for many
**Optical**	Gold Nanoparticles (for PTT/PDT), Quantum Dots (imaging), Upconversion NPs, Photoresponsive Hydrogels	Cancer Therapy (PTT/PDT), Bioimaging, Controlled Release, Optogenetics	High spatiotemporal precision, minimally invasive (NIR light), targeted effects	TRL 2–6
**Magnetic**	Iron Oxide Nanoparticles (IONPs/SPIONs)	MRI/MPI Contrast, Hyperthermia, Drug Targeting, Actuation	Deep tissue penetration, noninvasive remote control, Theranostics	TRL 4–9 for MRI, 3–6 for hyperthermia
**Ultrasound**	Microbubbles, Nanodroplets, Sonosensitizers	Contrast Imaging, Drug/Gene Delivery (Sonoporation), HIFU Therapy	Deep tissue penetration, noninvasive, real‐time imaging guidance, mechanical/thermal effects	TRL 5–9 for imaging, 3–7 for therapy

The range of applications and varying maturity levels across different stimulus types highlight both the immense therapeutic potential and the complex translational landscape facing this rapidly evolving field. In addition to the information provided in Table 2, there are significant challenges facing the successful translation of SRM technology into clinical/medical applications. SRM clearly represents a promising frontier in medical technology, yet its translation from laboratory concepts to clinical applications faces substantial hurdles across multiple domains. These materials, designed to respond predictably to electrical, optical, magnetic, or ultrasonic triggers, offer unprecedented opportunities for targeted drug delivery, tissue engineering, and minimally invasive therapies, but several critical challenges impede their widespread adoption, as detailed below.

#### 6.4.1 Clinical Translation

The transition from laboratory to clinical applications reveals fundamental disconnects between controlled research environments and human biology complexity. Laboratory studies employ simplified models that inadequately represent physiological complexity. In vivo conditions include dynamic blood flow, varying pH values, protein fouling, immune interactions, and mechanical stresses that are difficult to replicate. Protein corona formation can dramatically alter stimulus responsiveness, whereas inflammatory responses interfere with function. Patient population heterogeneity introduces variables, including genetic differences, disease severity, and comorbidities, that affect performance.

Achieving precise stimulus delivery in clinical settings is far more challenging than in laboratory conditions. Patient movement, breathing, and anatomical variations complicate targeting. Tissue depth and density affect stimulus penetration, particularly for optical and ultrasonic applications. Healthcare providers require training for safe, effective equipment operation, introducing the potential for human error and procedural variability. Determining optimal stimulus parameters requires understanding dose‒response relationships that vary significantly between patients. Unlike traditional drugs with established dosing methods, these materials require personalized protocols on the basis of tissue properties, disease state, and anatomy. The lack of standardized dosimetry methods complicates treatment planning and outcome comparisons.

#### 6.4.2 Biocompatibility and Safety Concerns

Another significant obstacle involves ensuring long‐term biocompatibility, which becomes exponentially more complex for stimuli‐responsive materials owing to their dynamic nature and potential for stimulus‐induced toxicity. Many stimuli‐responsive polymers and smart materials contain synthetic components that may trigger immune responses or exhibit cytotoxicity over extended periods. Unlike most traditional biomaterials with static properties, these materials undergo structural and chemical changes upon stimulus activation, potentially creating entirely new toxicological profiles that must be comprehensively evaluated.

Therefore, the fundamental challenge lies in characterizing materials that exist in multiple states depending on stimulus exposure. For example, a thermally responsive polymer may exhibit acceptable biocompatibility in its collapsed state but release toxic degradation products when heated above its transition temperature. Similarly, pH‐responsive materials may demonstrate excellent cytocompatibility under physiological conditions but become cytotoxic when triggered in the acidic microenvironments of inflamed or cancerous tissues. This dual‐state toxicology requires extensive testing under various activation conditions, significantly expanding the scope of safety assessments. This also applies to photodynamic materials, as their safety profile fundamentally changes upon light activation. The generation of reactive oxygen species during photoactivation may cause oxidative stress, DNA damage, and apoptosis, which are not detected in standard dark toxicity studies. The spatial and temporal distributions of these toxic effects must be carefully characterized to establish safe exposure limits and treatment protocols.

In addition, degradation products from SRM present unique safety concerns that extend beyond traditional biodegradable materials. Stimulus activation may accelerate degradation processes or create alternative degradation pathways that produce different metabolites than those observed under passive degradation conditions. For example, ultrasound‐triggered microbubbles may generate cavitation‐induced reactive species that catalyze unexpected degradation reactions, producing novel metabolites with unknown toxicological properties.

The dynamic nature of stimuli‐responsive materials also creates complex interactions with the immune system that differ significantly from those of static biomaterials. Stimulus‐induced conformational changes may expose new epitopes or alter protein adsorption patterns, triggering immune responses that would not occur with the unstimulated material. Repeated stimulus exposure raises concerns about sensitization reactions, where initial exposures prime the immune system for heightened responses to subsequent treatments. This is particularly relevant for materials requiring chronic or repeated administration, such as insulin delivery systems using glucose‐responsive polymers. The development of neutralizing antibodies against stimulus‐responsive drug carriers could compromise therapeutic efficacy and potentially create safety risks through immune complex formation.

Finally, establishing long‐term safety profiles for SRMs requires extended study periods that account for both chronic material presence and cumulative stimulus exposure effects. Traditional accelerated aging studies may not adequately predict long‐term behavior when materials undergo periodic activation cycles throughout their service life. The development of appropriate accelerated testing protocols that simulate realistic clinical use patterns while providing meaningful safety data within reasonable timeframes remains a significant challenge. The potential for unexpected long‐term effects emerges from the interaction between stimulus‐responsive materials and natural aging processes. Materials that remain stable under normal physiological conditions may undergo unexpected changes in elderly patients or those with age‐related alterations in tissue properties, pH, or enzymatic activity. These age‐related factors must be considered in safety assessment protocols to ensure adequate protection for diverse patient populations.

#### 6.4.3 Regulatory Pathway Complexity

The regulatory landscape for SRM presents one of the most formidable barriers to market entry, characterized by unprecedented complexity owing to the hybrid nature of these technologies and the absence of established regulatory precedents. The FDA's Office of Combination Products must determine the primary mode of action to assign the appropriate regulatory center, but this determination becomes contentious when the therapeutic effect depends equally on the material properties and external stimulus delivery. For example, magnetically responsive drug carriers require classification consideration of the nanoparticle formulation (FDA drug definition), the magnetic material (FDA device component), and the external magnetic field generator (FDA medical device). This tripartite nature often results in lengthy presubmission consultations and potential disagreements between different FDA centers.

The complexity increases when materials exhibit different regulatory profiles across various components. For example, a thermally responsive hydrogel drug carrier might be classified as a drug when considering active pharmaceutical ingredient release, as a device when evaluating the polymer matrix, and as requiring additional device submissions for the heating apparatus. Each component may require separate regulatory pathways with different timelines, data requirements, and approval criteria, creating coordination challenges and increasing regulatory costs.

Traditional regulatory frameworks rely on established mechanisms of action with well‐characterized safety and efficacy profiles. SRMs introduce entirely new therapeutic paradigms that often lack regulatory precedent. Agencies require a comprehensive mechanistic understanding, including a detailed characterization of stimulus‒response kinetics, dose‒response relationships for both the active agent and the stimulus, and potential off‐target effects from stimulus application. The mechanistic complexity extends to understanding how external stimuli interact with biological systems. For ultrasound‐responsive materials, regulators require data on acoustic dosimetry, tissue heating effects, cavitation potential, and mechanical bioeffects. Photodynamic applications must address phototoxicity, tissue penetration limitations, and the potential for photothermal damage. These novel mechanisms require the development of new testing methodologies and safety assessment protocols, as existing guidance documents provide limited relevant information.

Standard preclinical testing protocols often prove inadequate for stimuli‐responsive materials owing to their unique activation mechanisms. Traditional pharmacokinetic and toxicology studies must be modified to account for stimulus‐dependent drug release and activation. Agencies require a comprehensive characterization of material behavior under various stimulus conditions, including dose‒response relationships for both the therapeutic agent and the stimulus itself. Animal model selection becomes particularly challenging, as stimulus delivery systems must be appropriately scaled for different species while maintaining clinically relevant exposure conditions. The translational gap between animal models and human applications is amplified when anatomical differences affecting stimulus penetration and distribution are considered. Additionally, the lack of established biomarkers for stimulus‐responsive material efficacy complicates study endpoint selection and regulatory acceptance.

Genotoxicity testing requires special consideration when materials undergo stimulus‐induced structural changes that may create new chemical entities or degradation products. Standard battery testing may be insufficient if the activated form of the material has different toxicological properties than the inactive form does. Long‐term biocompatibility studies must account for potential cumulative effects from repeated stimulus exposures and chronic material implantation.

#### 6.4.4 Economic and Market Barriers

The economic challenges facing SRM translation create a complex web of financial barriers that significantly impact market entry and adoption. For example, the R&D pathway typically requires 10–15 years and hundreds of millions of dollars, depending on the complexity, of investment. Unlike traditional pharmaceuticals, these materials require custom manufacturing processes, specialized characterization equipment, and novel testing methodologies. The interdisciplinary nature inflates personnel costs, whereas specialized analytical equipment represents a substantial capital investment.

Clinical adoption requires significant infrastructure beyond the materials themselves. Hospitals must acquire stimulus‐delivery equipment, train personnel, and potentially redesign treatment spaces. For magnetic systems, facilities need electromagnetic shielding and specialized power. These infrastructure costs range from hundreds of thousands to millions per facility, creating barriers for smaller healthcare systems. Many applications target specialized conditions with small patient populations, limiting revenue potential. The conservative nature of medical practice means slow adoption, starting with academic centers before reaching community hospitals. This extended timeline delays returns on investment and complicates business case development.

## 7 Conclusions

This review demonstrates that SRMs represent a paradigm shift in biomedical engineering, offering unprecedented control over therapeutic interventions through electrical, optical, magnetic, and ultrasound activation mechanisms. Each stimulus modality has distinct advantages: electrical stimuli provide direct bioelectrical interface capabilities, optical stimuli deliver unmatched spatial precision, magnetic stimuli enable deep tissue penetration, and ultrasound stimuli combine excellent penetration with established safety profiles. The diversity of responsive materials, from conductive polymers and photoresponsive nanoparticles to iron oxide nanocomposites and microbubbles, illustrates how materials science innovations are creating new possibilities for precision medicine. However, the selection of appropriate stimulus‒material combinations requires systematic evaluation of tissue penetration, spatial resolution, temporal control, and safety considerations specific to each clinical application. The framework presented here provides clinicians and researchers with essential guidance for matching stimulus modalities to therapeutic objectives, moving beyond trial–and‐error approaches toward evidence‐based selection criteria.

The interdisciplinary nature of this field continues to drive rapid innovation, bringing together expertise from materials science, biomedical engineering, clinical medicine, and regulatory science to overcome complex translation challenges. Near‐term clinical applications show particular promise where existing technologies have established regulatory pathways: magnetic‐responsive contrast agents are revolutionizing diagnostic imaging, US‐responsive microbubbles are expanding therapeutic capabilities, and electrically responsive neural interfaces are approaching clinical reality. Simultaneously, long‐term revolutionary potential has extended to remotely controlled drug factories, light‐activated genetic switches, and magnetically guided cellular reprogramming for regenerative medicine. These transformative applications will require continued interdisciplinary collaboration to address fundamental challenges in biocompatibility, long‐term stability, and precise stimulus delivery in biological environments.

Despite significant technical advances, the path from laboratory innovation to clinical implementation remains complex, requiring navigation of regulatory frameworks that often lack precedent for these hybrid technologies. The technology readiness level analysis reveals substantial variation across stimulus types, with magnetic systems achieving the highest clinical maturity, whereas electrical and optical systems remain in earlier development stages. Success in translating these materials will depend on continued investment in fundamental research, the development of standardized testing protocols, and the establishment of clear regulatory pathways for stimuli‐responsive systems. As these challenges are systematically addressed through interdisciplinary collaboration, SRMs have the potential to transform healthcare delivery by enabling truly personalized, remotely controlled, and minimally invasive therapeutic interventions. The convergence of advancing material capabilities with improving stimulus delivery technologies suggests that the most impactful applications may emerge from integrated systems combining multiple stimulus modalities, creating adaptive therapeutic platforms with unprecedented sophistication.

## Conflict of Interest

The authors declare no conflict of interest.
